# 4-[2-(4-Hy­droxy­phen­yl)eth­yl]-3-propyl-1*H*-1,2,4-triazol-5(4*H*)-one

**DOI:** 10.1107/S1600536812019447

**Published:** 2012-05-05

**Authors:** Sema Öztürk Yildirim, Ray J. Butcher, Dilek Ünlüer, Yavuz Köysal

**Affiliations:** a Chemistry Department, Howard University, Washington, DC 20059, USA; c Department of Chemistry, Karadeniz Technical University, Trabzon TR-61080, Turkey; b Department of Physics, Faculty of Sciences, Erciyes University, 38039 Kayseri, Turkey; d Yeşilyurt Demir Çelik Vocational School, Ondokuz Mayis University, Samsun, Turkey

## Abstract

The asymmetric unit of the title compound, C_13_H_17_N_3_O_2_, contains eight crystallographically independent mol­ecules. The planes of the benzene and triazole rings in the eight mol­ecules make dihedral angles of 5.53 (13), 9.33 (13), 19.28 (11), 17.36 (8), 12.84 (12), 8.03 (8), 19.97 (11), and 7.98 (8)°. The eight mol­ecules in the asymmetric unit are linked by inter­molecular O—H⋯O and N—H⋯O hydrogen bonds, forming a three-dimensional network.

## Related literature

For the anti­viral activity of triazoles, see: Sancak *et al.* (2012 ▶); Gurumoorthy *et al.* (2011 ▶). For the synthesis of anti­biotics, fungicides, herbicides and plant growth hormone insulators, see: Ünver *et al.* (2006 ▶, 2011 ▶). For potentially good corrosion inhibitions, see: Lebrini *et al.* (2008 ▶). For bond lengths in related structures, see: Öztürk *et al.* (2004*a* ▶,*b* ▶); Akkurt *et al.* (2004 ▶). For standard bond lengths, see: Allen *et al.* (1987 ▶).
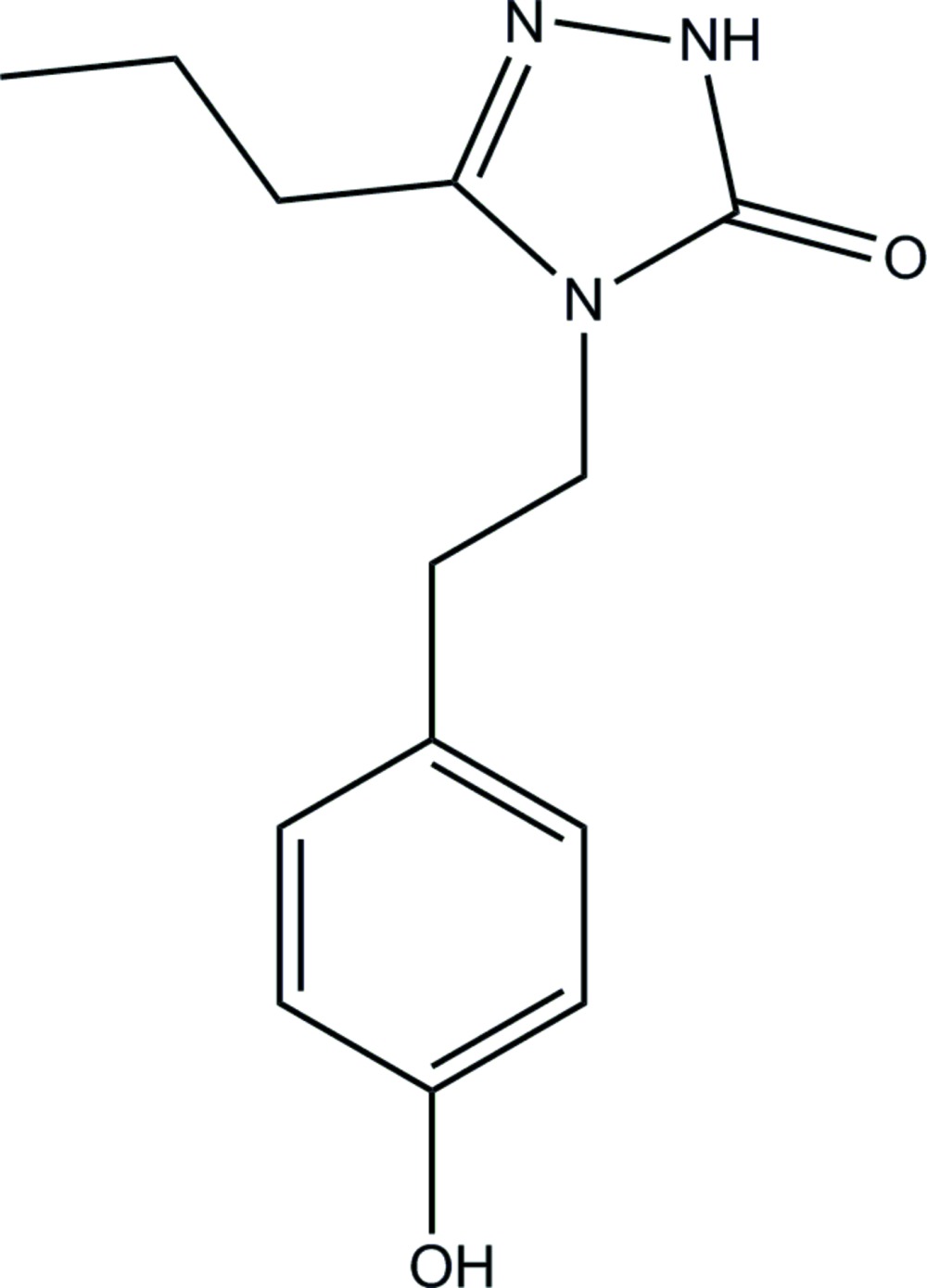


## Experimental

### 

#### Crystal data

C_13_H_17_N_3_O_2_
*M* *_r_* = 247.30
Monoclinic, 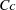

*a* = 26.2670 (4) Å
*b* = 26.3371 (4) Å
*c* = 15.9037 (3) Å
β = 111.8715 (19)°
*V* = 10210.2 (3) Å^3^
*Z* = 32
Cu *K*α radiation
μ = 0.72 mm^−1^
*T* = 123 K
0.55 × 0.40 × 0.20 mm

#### Data collection

Oxford Diffraction Gemini-R diffractometer
Absorption correction: multi-scan (*CrysAlis RED*; Oxford Diffraction, 2007 ▶) *T*_min_ = 0.756, *T*_max_ = 0.865
20766 measured reflections
12229 independent reflections
11392 reflections with *I* > 2σ(*I*)
*R*_int_ = 0.028

#### Refinement

*R*[*F* ^2^ > 2σ(*F* ^2^)] = 0.040
*wR*(*F* ^2^) = 0.108
*S* = 1.02
12229 reflections
1313 parameters
2 restraints
H-atom parameters constrained
Δρ_max_ = 0.56 e Å^−3^
Δρ_min_ = −0.23 e Å^−3^
Absolute structure: Flack (1983 ▶), 1607 Friedel pairs
Flack parameter: −0.08 (12)

### 

Data collection: *CrysAlis PRO* (Oxford Diffraction, 2007 ▶); cell refinement: *CrysAlis PRO*; data reduction: *CrysAlis PRO*; program(s) used to solve structure: *SHELXS97* (Sheldrick, 2008 ▶); program(s) used to refine structure: *SHELXL97* (Sheldrick, 2008 ▶); molecular graphics: *SHELXTL* (Sheldrick, 2008 ▶); software used to prepare material for publication: *SHELXTL*.

## Supplementary Material

Crystal structure: contains datablock(s) I, global. DOI: 10.1107/S1600536812019447/hg5217sup1.cif

Structure factors: contains datablock(s) I. DOI: 10.1107/S1600536812019447/hg5217Isup2.hkl

Supplementary material file. DOI: 10.1107/S1600536812019447/hg5217Isup3.cml

Additional supplementary materials:  crystallographic information; 3D view; checkCIF report

## Figures and Tables

**Table 1:** Hydrogen-bond geometry (Å, °)

*D*—H⋯*A*	*D*—H	H⋯*A*	*D*⋯*A*	*D*—H⋯*A*
O2*A*—H2*A*⋯O1*H*	0.82	1.90	2.718 (3)	174
O2*C*—H2*C*⋯O1*E*	0.82	1.90	2.717 (3)	172
O2*G*—H2*G*⋯O1*C*	0.82	1.89	2.701 (3)	172
O2*H*—H2*H*⋯O1*D*	0.82	1.94	2.752 (3)	174
N2*B*—H2*BA*⋯O1*C*	0.86	1.97	2.821 (3)	169
N2*C*—H2*CA*⋯O1*B*	0.86	1.97	2.825 (3)	174
N2*F*—H2*FA*⋯O1*H*	0.86	1.93	2.777 (3)	166
N2*H*—H2*HA*⋯O1*F*	0.86	2.00	2.840 (3)	167
O2*B*—H2*B*⋯O1*G*^i^	0.82	1.93	2.748 (3)	177
O2*D*—H2*D*⋯O1*F*^i^	0.82	1.87	2.689 (3)	178
O2*E*—H2*E*⋯O1*B*^ii^	0.82	1.97	2.773 (3)	165
O2*F*—H2*F*⋯O1*A*^ii^	0.82	1.92	2.711 (3)	162
N2*A*—H2*AA*⋯O1*D*^iii^	0.86	1.94	2.788 (3)	170
N2*E*—H2*EA*⋯O1*G*^iii^	0.86	2.03	2.873 (3)	168
N2*D*—H2*DA*⋯O1*A*^iv^	0.86	1.95	2.809 (3)	173
N2*G*—H2*GA*⋯O1*E*^iv^	0.86	1.96	2.807 (3)	168

Symmetry codes: (i) 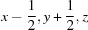; (ii) 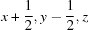; (iii) 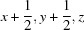; (iv) 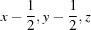.

**Fig. 1.**
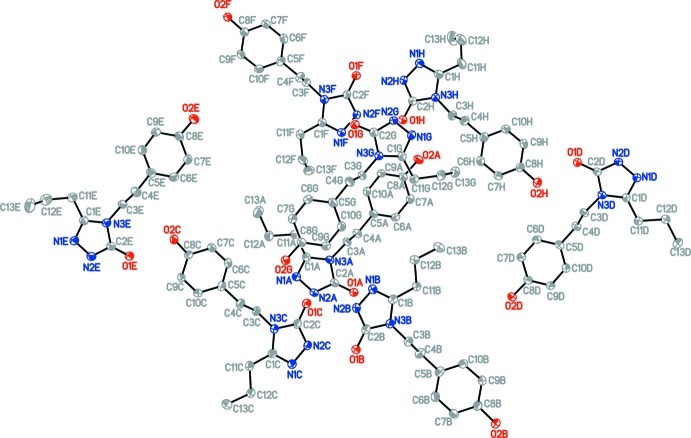
The eight independent molecules in the asymmetric unit of the title compound, showing the atom-numbering scheme. Displacement ellipsoids are drawn at the 50% probability level. H atoms have been omitted for clarity.

**Fig. 2.**
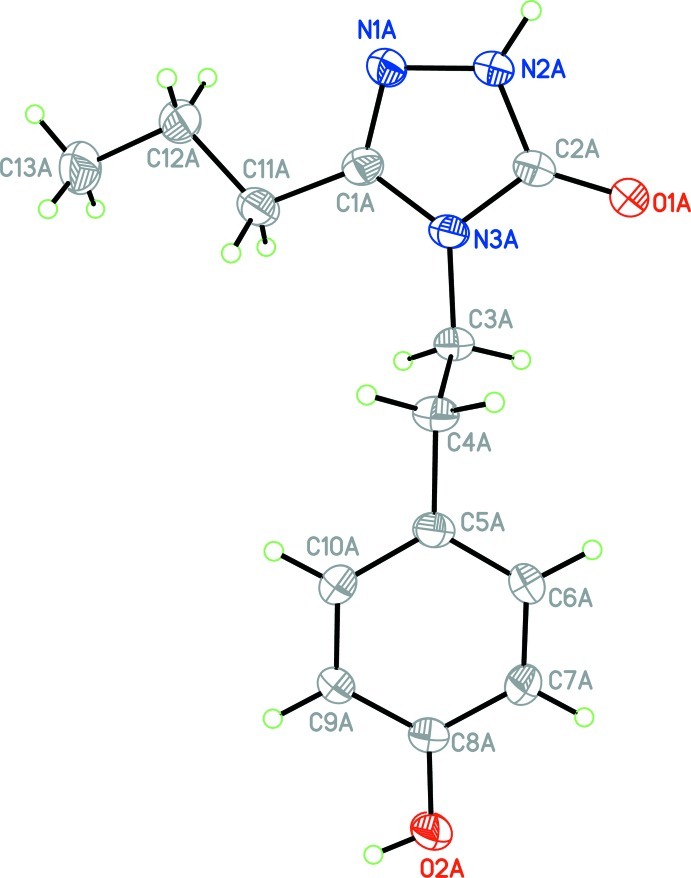
One of the eight independent molecules in the asymmetric unit to show clearly the atomic labelling (molecule A). The other seven molecules are labelled in a similar manner.

**Fig. 3.**
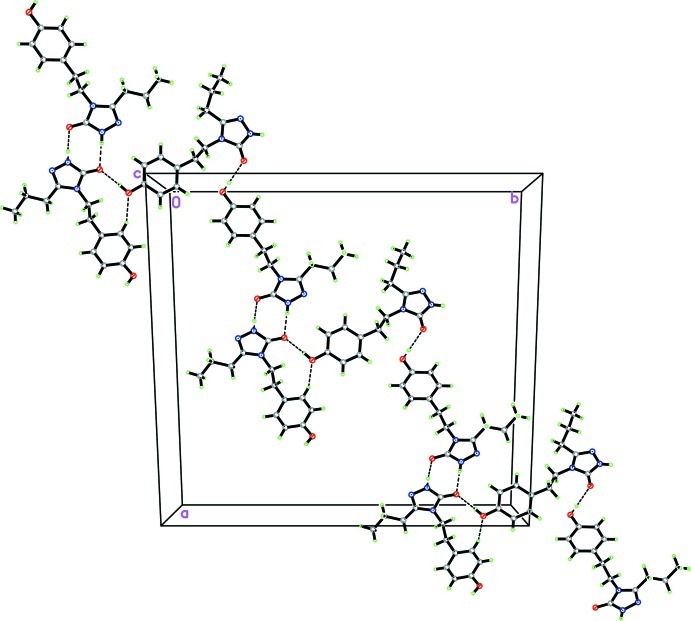
The crystal packing of the title compound viewed along the c axis, showing the formation of hydrogen bonded dimers further linked by additional hydrogen bonding. The hydrogen bonds as dashed lines.

## supplementary crystallographic information

### Comment

During the past two decades, triazole and its derivatives represent an important class of heterocycles. The arrangement of 3 basic nitrogen atoms in the triazole ring induces antiviral activities in the compounds containing this ring (Sancak *et al.*, 2012; Gurumoorthy *et al.*, 2011). They are of biological importance and are used in the synthesis of drugs such as antibiotics, fungicides, herbicides, and plant growth hormone insulators (Ünver *et al.*, 2011; Ünver *et al.*, 2006) and are also potentially good corrosion inhibitors (Lebrini *et al.*, 2008).

Eight independent molecules of the title compound (I) in the asymmetric unit are shown in Fig. 1 and labelled as A to H. The bond lengths and angles in the eight molecules have normal values (Öztürk *et al.*, 2004*a*; Öztürk *et al.*, 2004*b*; Akkurt *et al.*, 2004; Allen *et al.*, 1987). The phenyl and triazol rings in molecules (A - H) make dihedral angles of 5.53 (13) °, 9.33 (13) °, 19.28 (11) °, 17.36 (08) °, 12.84 (12) °, 8.03 (08) °, 19.97 (11) ° and 7.98 (08) ° to each other, respectively.

The propyl group on the bridging C1 atom position and takes a zigzag form in eight molecules forming torsion angles of 178.27 (25) ° in molecule A [179.63 (26) ° in B; 176.97 (25) ° in C; -174.29 (23) ° in D; 72.54 (40) ° in E; 73.56 (31) ° in F; -73.09 (30) ° in G and -67.50 (40)° in H].

The oxygen atoms are coplanar with the benzene and triazol rings in all molecules. The major difference in conformation for the 8 molecules occurs for the orientation of the propyl group with respect to the triazole ring. For molecules A, B, C, D, E, F, G and H these values are 6.5 (4), 0.4 (4), 1.9 (4), 13.7 (4), 12.2 (4), 0.9 (4), -8.5 (4) and -16.3 (5), respectively.

In the crystal structure (Fig. 3), the molecules are linked through O–H···O and N–H···O intra- and intermolecular hydrogen bonds (Table 1) to form a three-dimensional network.

### Experimental

A solution of 4-(4-metoksifeniletil)-5-propil-2*H*-1,2,4-triazol-3(4*H*)-on (10 mmol) in chloroform (100 ml) was added to a solution of boron tribromide (10 mmol) in chloroform (200 ml) at 273 K. The reaction mixture was poured into ice containing sufficient 50% sodium hydroxide to attain a pH of 10. The addition of concentrated sulfuric acid provided a precipitate that was extracted into ether. The combined organic extract was washed with water and brine, and then dried and concentrated in a vacuum to obtain compounds. The crude product was recrystallized using ethyl acetate and petroleum ether (1:2) to afford the desired compound. Yield: 130 mg (40%). Mp: 455–456 K.

### Refinement

H atoms were positioned geometrically [C–H = 0.93–0.97 Å; N–H = 0.86 Å and O–H = 0.82 Å] and refined using a riding model with *U*_iso_(H) = 1.2*_U_*_eq_(C,*N*) and 1.5*U*_eq_(O). For some of the strongest reflections detector saturation was observed as F_o_ was significantly less than F_c_.

### Crystal data

**Table:**

C_13_H_17_N_3_O_2_	*F*(000) = 4224
*M**_r_* = 247.30	*D*_x_ = 1.287 Mg m^−^^3^
Monoclinic, *C**c*	Cu *K*α radiation, λ = 1.54184 Å
Hall symbol: C -2yc	Cell parameters from 9884 reflections
*a* = 26.2670 (4) Å	θ = 3.0–75.5°
*b* = 26.3371 (4) Å	µ = 0.72 mm^−^^1^
*c* = 15.9037 (3) Å	*T* = 123 K
β = 111.8715 (19)°	Plate, colorless
*V* = 10210.2 (3) Å^3^	0.55 × 0.40 × 0.20 mm
*Z* = 32	

### Data collection

**Table:**

Oxford Diffraction Gemini-R diffractometer	12229 independent reflections
Radiation source: Enhance (Cu) X-ray Source	11392 reflections with *I* > 2σ(*I*)
Graphite monochromator	*R*_int_ = 0.028
Detector resolution: 10.5081 pixels mm^-1^	θ_max_ = 75.7°, θ_min_ = 3.3°
ω scans	*h* = −32→29
Absorption correction: multi-scan (*CrysAlis RED*; Oxford Diffraction, 2007)	*k* = −28→33
*T*_min_ = 0.756, *T*_max_ = 0.865	*l* = −16→19
20766 measured reflections	

### Refinement

**Table:**

Refinement on *F*^2^	Secondary atom site location: difference Fourier map
Least-squares matrix: full	Hydrogen site location: inferred from neighbouring sites
*R*[*F*^2^ > 2σ(*F*^2^)] = 0.040	H-atom parameters constrained
*wR*(*F*^2^) = 0.108	*w* = 1/[σ^2^(*F*_o_^2^) + (0.0694*P*)^2^ + 1.6747*P*] where *P* = (*F*_o_^2^ + 2*F*_c_^2^)/3
*S* = 1.02	(Δ/σ)_max_ < 0.001
12229 reflections	Δρ_max_ = 0.56 e Å^−^^3^
1313 parameters	Δρ_min_ = −0.23 e Å^−^^3^
2 restraints	Absolute structure: Flack (1983), 1607 Friedel pairs
Primary atom site location: structure-invariant direct methods	Flack parameter: −0.08 (12)

### Special details

**Table:**

Experimental. CrysAlis RED, (Oxford Diffraction, 2007) Empirical absorption correction using spherical harmonics, implemented in SCALE3 ABSPACK scaling algorithm. (Clark & Reid, 1995).
Geometry. All e.s.d.'s (except the e.s.d. in the dihedral angle between two l.s. planes) are estimated using the full covariance matrix. The cell e.s.d.'s are taken into account individually in the estimation of e.s.d.'s in distances, angles and torsion angles; correlations between e.s.d.'s in cell parameters are only used when they are defined by crystal symmetry. An approximate (isotropic) treatment of cell e.s.d.'s is used for estimating e.s.d.'s involving l.s. planes.
Refinement. Refinement of *F*^2^ against ALL reflections. The weighted *R*-factor *wR* and goodness of fit *S* are based on *F*^2^, conventional *R*-factors *R* are based on *F*, with *F* set to zero for negative *F*^2^. The threshold expression of *F*^2^ > σ(*F*^2^) is used only for calculating *R*-factors(gt) *etc*. and is not relevant to the choice of reflections for refinement. *R*-factors based on *F*^2^ are statistically about twice as large as those based on *F*, and *R*- factors based on ALL data will be even larger.

### Fractional atomic coordinates and isotropic or equivalent isotropic displacement parameters (Å^2^)

**Table:**

	*x*	*y*	*z*	*U*_iso_*/*U*_eq_	
O1A	0.32042 (7)	0.63059 (7)	0.53991 (14)	0.0313 (4)	
O2A	0.23969 (9)	0.31435 (7)	0.54837 (16)	0.0379 (5)	
H2A	0.2556	0.2896	0.5384	0.057*	
O1B	0.32993 (7)	0.74837 (7)	0.78314 (14)	0.0318 (4)	
O2B	0.01487 (8)	0.83745 (7)	0.78241 (15)	0.0354 (4)	
H2B	−0.0127	0.8198	0.7698	0.053*	
O1C	0.44855 (7)	0.67523 (7)	0.75349 (14)	0.0329 (4)	
O2C	0.76009 (7)	0.59803 (7)	0.73340 (14)	0.0317 (4)	
H2C	0.7853	0.6168	0.7350	0.048*	
O1D	−0.10864 (7)	0.25363 (7)	0.52936 (14)	0.0325 (4)	
O2D	−0.02729 (8)	0.57050 (7)	0.52025 (16)	0.0395 (5)	
H2D	−0.0461	0.5950	0.5219	0.059*	
O1E	0.85070 (7)	0.65276 (7)	0.74818 (13)	0.0302 (4)	
O2E	0.77893 (8)	0.33691 (7)	0.80454 (15)	0.0358 (4)	
H2E	0.7983	0.3121	0.8074	0.054*	
O1F	0.41178 (7)	0.15199 (7)	0.52337 (14)	0.0324 (4)	
O2F	0.73204 (8)	0.07192 (7)	0.52117 (17)	0.0397 (5)	
H2F	0.7578	0.0919	0.5368	0.059*	
O1G	0.42183 (8)	0.27873 (7)	0.73332 (15)	0.0349 (4)	
O2G	0.51778 (8)	0.59517 (7)	0.79702 (15)	0.0356 (4)	
H2G	0.4960	0.6188	0.7880	0.053*	
O1H	0.28519 (8)	0.22996 (7)	0.50699 (15)	0.0362 (4)	
O2H	−0.04974 (8)	0.30837 (8)	0.44907 (16)	0.0399 (5)	
H2H	−0.0694	0.2935	0.4708	0.060*	
N1A	0.45579 (9)	0.62266 (8)	0.55514 (15)	0.0290 (4)	
N2A	0.40789 (9)	0.64889 (8)	0.54266 (16)	0.0288 (4)	
H2AA	0.4042	0.6811	0.5329	0.035*	
N3A	0.39078 (8)	0.57114 (8)	0.56143 (14)	0.0255 (4)	
N1B	0.31491 (9)	0.61776 (8)	0.74832 (16)	0.0310 (5)	
N2B	0.34211 (9)	0.66325 (9)	0.75468 (17)	0.0320 (5)	
H2BA	0.3727	0.6660	0.7470	0.038*	
N3B	0.27049 (8)	0.68123 (8)	0.78210 (15)	0.0273 (4)	
N1C	0.45086 (8)	0.80713 (8)	0.74037 (15)	0.0278 (4)	
N2C	0.42766 (8)	0.76158 (8)	0.75037 (15)	0.0277 (4)	
H2CA	0.3969	0.7593	0.7576	0.033*	
N3C	0.50306 (8)	0.74212 (8)	0.73528 (14)	0.0250 (4)	
N1D	−0.23985 (9)	0.26324 (8)	0.53437 (16)	0.0296 (5)	
N2D	−0.19493 (9)	0.23628 (8)	0.53301 (17)	0.0301 (5)	
H2DA	−0.1934	0.2037	0.5330	0.036*	
N3D	−0.17309 (8)	0.31451 (8)	0.53410 (14)	0.0259 (4)	
N1E	0.97940 (9)	0.64425 (9)	0.73396 (17)	0.0320 (5)	
N2E	0.93372 (9)	0.67033 (9)	0.73233 (16)	0.0309 (5)	
H2EA	0.9299	0.7026	0.7248	0.037*	
N3E	0.91736 (9)	0.59232 (8)	0.75091 (15)	0.0269 (4)	
N1F	0.41392 (9)	0.28268 (9)	0.49190 (18)	0.0336 (5)	
N2F	0.38850 (9)	0.23736 (9)	0.49803 (18)	0.0333 (5)	
H2FA	0.3545	0.2350	0.4913	0.040*	
N3F	0.47198 (8)	0.21841 (8)	0.52262 (14)	0.0274 (4)	
N1G	0.30703 (9)	0.28325 (9)	0.79292 (17)	0.0319 (5)	
N2G	0.34766 (9)	0.25805 (9)	0.77351 (18)	0.0338 (5)	
H2GA	0.3508	0.2255	0.7745	0.041*	
N3G	0.36132 (8)	0.33700 (8)	0.75877 (15)	0.0262 (4)	
N1H	0.27545 (9)	0.09883 (9)	0.52067 (17)	0.0329 (5)	
N2H	0.30407 (8)	0.14291 (8)	0.52342 (15)	0.0283 (4)	
H2HA	0.3384	0.1438	0.5324	0.034*	
N3H	0.22160 (9)	0.16590 (8)	0.50043 (15)	0.0283 (4)	
C1A	0.44423 (10)	0.57584 (10)	0.56539 (17)	0.0273 (5)	
C2A	0.36784 (10)	0.61875 (10)	0.54741 (17)	0.0264 (5)	
C3A	0.36292 (10)	0.52515 (9)	0.57252 (17)	0.0261 (5)	
H3AA	0.3382	0.5337	0.6028	0.031*	
H3AB	0.3900	0.5015	0.6109	0.031*	
C4A	0.33048 (10)	0.49927 (10)	0.48268 (17)	0.0283 (5)	
H4AA	0.3013	0.5216	0.4459	0.034*	
H4AB	0.3545	0.4927	0.4500	0.034*	
C5A	0.30604 (10)	0.44984 (9)	0.49823 (17)	0.0258 (5)	
C6A	0.26004 (11)	0.44895 (10)	0.52255 (19)	0.0304 (5)	
H6AA	0.2434	0.4794	0.5276	0.037*	
C7A	0.23876 (11)	0.40359 (10)	0.53929 (19)	0.0315 (5)	
H7AA	0.2082	0.4039	0.5556	0.038*	
C8A	0.26274 (10)	0.35775 (10)	0.53183 (18)	0.0277 (5)	
C9A	0.30856 (10)	0.35782 (10)	0.50727 (18)	0.0281 (5)	
H9AA	0.3250	0.3273	0.5023	0.034*	
C10A	0.32942 (10)	0.40309 (10)	0.49044 (18)	0.0280 (5)	
H10A	0.3597	0.4026	0.4735	0.034*	
C11A	0.48128 (10)	0.53146 (10)	0.57520 (19)	0.0316 (5)	
H11A	0.4654	0.5093	0.5232	0.038*	
H11B	0.4838	0.5124	0.6288	0.038*	
C12A	0.53880 (11)	0.54654 (12)	0.5828 (2)	0.0391 (6)	
H12A	0.5548	0.5693	0.6339	0.047*	
H12B	0.5368	0.5644	0.5284	0.047*	
C13A	0.57514 (12)	0.49947 (13)	0.5956 (3)	0.0493 (8)	
H13A	0.6118	0.5097	0.6039	0.074*	
H13B	0.5608	0.4782	0.5430	0.074*	
H13C	0.5757	0.4810	0.6479	0.074*	
C1B	0.27145 (11)	0.63014 (10)	0.76452 (18)	0.0289 (5)	
C2B	0.31625 (10)	0.70283 (10)	0.77401 (18)	0.0287 (5)	
C3B	0.22882 (10)	0.70967 (10)	0.80311 (17)	0.0275 (5)	
H3BA	0.2461	0.7381	0.8422	0.033*	
H3BB	0.2127	0.6878	0.8356	0.033*	
C4B	0.18385 (11)	0.72939 (11)	0.71778 (18)	0.0325 (5)	
H4BA	0.2000	0.7516	0.6858	0.039*	
H4BB	0.1671	0.7010	0.6783	0.039*	
C5B	0.14000 (10)	0.75817 (11)	0.73858 (17)	0.0292 (5)	
C6B	0.13950 (10)	0.81096 (10)	0.74132 (17)	0.0285 (5)	
H6BA	0.1677	0.8291	0.7334	0.034*	
C7B	0.09767 (10)	0.83679 (10)	0.75571 (18)	0.0285 (5)	
H7BA	0.0976	0.8721	0.7562	0.034*	
C8B	0.05551 (10)	0.81020 (10)	0.76943 (17)	0.0275 (5)	
C9B	0.05670 (10)	0.75734 (10)	0.77074 (18)	0.0282 (5)	
H9BA	0.0295	0.7391	0.7819	0.034*	
C10B	0.09844 (10)	0.73217 (10)	0.75530 (18)	0.0294 (5)	
H10B	0.0989	0.6969	0.7561	0.035*	
C11B	0.22785 (10)	0.59363 (10)	0.7646 (2)	0.0314 (5)	
H11C	0.1930	0.6043	0.7197	0.038*	
H11D	0.2247	0.5946	0.8235	0.038*	
C12B	0.23949 (11)	0.53921 (10)	0.7442 (2)	0.0339 (6)	
H12C	0.2743	0.5283	0.7889	0.041*	
H12D	0.2422	0.5379	0.6851	0.041*	
C13B	0.19416 (14)	0.50327 (12)	0.7457 (3)	0.0512 (8)	
H13D	0.2019	0.4695	0.7311	0.077*	
H13E	0.1596	0.5143	0.7019	0.077*	
H13F	0.1925	0.5034	0.8049	0.077*	
C1C	0.49598 (10)	0.79413 (10)	0.73145 (16)	0.0258 (5)	
C2C	0.45827 (9)	0.72142 (10)	0.74753 (17)	0.0266 (5)	
C3C	0.54834 (9)	0.71313 (10)	0.72567 (17)	0.0258 (5)	
H3CA	0.5621	0.7314	0.6856	0.031*	
H3CB	0.5344	0.6807	0.6977	0.031*	
C4C	0.59583 (10)	0.70378 (10)	0.81609 (17)	0.0278 (5)	
H4CA	0.6094	0.7360	0.8456	0.033*	
H4CB	0.5830	0.6839	0.8556	0.033*	
C5C	0.64181 (9)	0.67558 (10)	0.79965 (16)	0.0265 (5)	
C6C	0.63595 (10)	0.62491 (10)	0.77457 (18)	0.0286 (5)	
H6CA	0.6045	0.6075	0.7718	0.034*	
C7C	0.67588 (10)	0.59966 (10)	0.75362 (18)	0.0283 (5)	
H7CA	0.6710	0.5657	0.7367	0.034*	
C8C	0.72312 (10)	0.62466 (9)	0.75761 (16)	0.0251 (5)	
C9C	0.73047 (9)	0.67531 (10)	0.78506 (17)	0.0255 (5)	
H9CA	0.7625	0.6923	0.7899	0.031*	
C10C	0.68988 (10)	0.70044 (9)	0.80514 (17)	0.0262 (5)	
H10C	0.6948	0.7344	0.8225	0.031*	
C11C	0.53753 (10)	0.83008 (10)	0.72183 (18)	0.0295 (5)	
H11E	0.5412	0.8238	0.6643	0.035*	
H11F	0.5728	0.8232	0.7695	0.035*	
C12C	0.52325 (10)	0.88555 (10)	0.7265 (2)	0.0336 (6)	
H12E	0.5179	0.8919	0.7827	0.040*	
H12F	0.4892	0.8933	0.6767	0.040*	
C13C	0.56868 (13)	0.91997 (12)	0.7213 (3)	0.0498 (8)	
H13G	0.5573	0.9548	0.7187	0.075*	
H13H	0.5759	0.9119	0.6680	0.075*	
H13I	0.6014	0.9149	0.7740	0.075*	
C1D	−0.22557 (10)	0.31056 (10)	0.53420 (17)	0.0248 (5)	
C2D	−0.15421 (10)	0.26607 (10)	0.53166 (18)	0.0277 (5)	
C3D	−0.14020 (10)	0.36041 (9)	0.54415 (17)	0.0258 (5)	
H3DA	−0.1017	0.3514	0.5703	0.031*	
H3DB	−0.1477	0.3832	0.5861	0.031*	
C4D	−0.15130 (11)	0.38808 (10)	0.45537 (17)	0.0286 (5)	
H4DA	−0.1411	0.3666	0.4146	0.034*	
H4DB	−0.1901	0.3956	0.4269	0.034*	
C5D	−0.11882 (10)	0.43685 (10)	0.47221 (17)	0.0275 (5)	
C6D	−0.06162 (11)	0.43622 (10)	0.49891 (18)	0.0301 (5)	
H6DA	−0.0434	0.4053	0.5062	0.036*	
C7D	−0.03165 (11)	0.48107 (10)	0.51469 (19)	0.0311 (5)	
H7DA	0.0063	0.4800	0.5326	0.037*	
C8D	−0.05853 (11)	0.52757 (10)	0.50371 (18)	0.0294 (5)	
C9D	−0.11529 (10)	0.52878 (10)	0.47624 (18)	0.0284 (5)	
H9DA	−0.1336	0.5597	0.4678	0.034*	
C10D	−0.14453 (10)	0.48368 (10)	0.46152 (18)	0.0284 (5)	
H10D	−0.1825	0.4849	0.4440	0.034*	
C11D	−0.26104 (10)	0.35525 (10)	0.53059 (18)	0.0276 (5)	
H11G	−0.2721	0.3707	0.4711	0.033*	
H11H	−0.2399	0.3802	0.5747	0.033*	
C12D	−0.31203 (11)	0.34160 (10)	0.54950 (19)	0.0314 (5)	
H12G	−0.3011	0.3294	0.6113	0.038*	
H12H	−0.3314	0.3143	0.5093	0.038*	
C13D	−0.35046 (12)	0.38672 (12)	0.5362 (2)	0.0399 (6)	
H13J	−0.3813	0.3769	0.5511	0.060*	
H13K	−0.3631	0.3976	0.4742	0.060*	
H13L	−0.3312	0.4141	0.5750	0.060*	
C1E	0.96840 (10)	0.59709 (10)	0.74532 (18)	0.0297 (5)	
C2E	0.89555 (10)	0.64016 (10)	0.74376 (17)	0.0272 (5)	
C3E	0.89208 (10)	0.54646 (9)	0.77215 (18)	0.0275 (5)	
H3EA	0.8651	0.5569	0.7970	0.033*	
H3EB	0.9202	0.5273	0.8186	0.033*	
C4E	0.86432 (12)	0.51184 (11)	0.69162 (18)	0.0332 (6)	
H4EA	0.8346	0.5299	0.6459	0.040*	
H4EB	0.8905	0.5016	0.6651	0.040*	
C5E	0.84198 (11)	0.46534 (10)	0.72235 (18)	0.0297 (5)	
C6E	0.78989 (11)	0.46506 (10)	0.72721 (19)	0.0327 (6)	
H6EA	0.7684	0.4942	0.7112	0.039*	
C7E	0.76950 (11)	0.42278 (11)	0.75502 (19)	0.0320 (6)	
H7EA	0.7345	0.4237	0.7569	0.038*	
C8E	0.80117 (11)	0.37835 (10)	0.78056 (18)	0.0284 (5)	
C9E	0.85391 (10)	0.37868 (10)	0.77885 (18)	0.0296 (5)	
H9EA	0.8760	0.3501	0.7970	0.035*	
C10E	0.87334 (10)	0.42162 (10)	0.75011 (17)	0.0290 (5)	
H10E	0.9086	0.4211	0.7494	0.035*	
C11E	1.00545 (11)	0.55273 (11)	0.7505 (2)	0.0373 (6)	
H11I	0.9898	0.5325	0.6959	0.045*	
H11J	1.0075	0.5315	0.8016	0.045*	
C12E	1.06329 (14)	0.56939 (15)	0.7613 (3)	0.0560 (9)	
H12I	1.0826	0.5406	0.7494	0.067*	
H12J	1.0606	0.5952	0.7164	0.067*	
C13E	1.09669 (14)	0.59040 (17)	0.8551 (3)	0.0645 (11)	
H13M	1.1343	0.5940	0.8615	0.097*	
H13N	1.0944	0.5675	0.9005	0.097*	
H13O	1.0824	0.6230	0.8623	0.097*	
C1F	0.46387 (10)	0.26985 (10)	0.50665 (18)	0.0286 (5)	
C2F	0.42275 (10)	0.19774 (10)	0.51565 (17)	0.0276 (5)	
C3F	0.52270 (10)	0.18953 (10)	0.54213 (18)	0.0275 (5)	
H3FA	0.5224	0.1602	0.5787	0.033*	
H3FB	0.5538	0.2106	0.5768	0.033*	
C4F	0.52939 (11)	0.17203 (11)	0.45564 (18)	0.0332 (6)	
H4FA	0.4997	0.1490	0.4233	0.040*	
H4FB	0.5269	0.2012	0.4171	0.040*	
C5F	0.58369 (10)	0.14547 (11)	0.47461 (18)	0.0305 (5)	
C6F	0.58814 (11)	0.09310 (11)	0.4788 (2)	0.0382 (6)	
H6FA	0.5571	0.0736	0.4705	0.046*	
C7F	0.63817 (12)	0.06896 (11)	0.4952 (2)	0.0392 (6)	
H7FA	0.6402	0.0337	0.4976	0.047*	
C8F	0.68486 (11)	0.09739 (10)	0.50772 (19)	0.0314 (5)	
C9F	0.68148 (11)	0.15013 (10)	0.50604 (19)	0.0304 (5)	
H9FA	0.7127	0.1697	0.5159	0.036*	
C10F	0.63112 (11)	0.17344 (10)	0.48944 (19)	0.0309 (5)	
H10F	0.6291	0.2087	0.4882	0.037*	
C11F	0.50771 (11)	0.30590 (11)	0.5055 (2)	0.0328 (6)	
H11K	0.5214	0.2951	0.4595	0.039*	
H11L	0.5380	0.3046	0.5636	0.039*	
C12F	0.48721 (12)	0.36056 (11)	0.4862 (2)	0.0385 (6)	
H12K	0.5136	0.3803	0.4704	0.046*	
H12L	0.4528	0.3608	0.4343	0.046*	
C13F	0.47866 (12)	0.38548 (12)	0.5654 (2)	0.0445 (7)	
H13P	0.4619	0.4181	0.5473	0.067*	
H13Q	0.5134	0.3896	0.6143	0.067*	
H13R	0.4553	0.3645	0.5850	0.067*	
C1G	0.31662 (10)	0.33080 (10)	0.78410 (18)	0.0274 (5)	
C2G	0.38132 (10)	0.28937 (10)	0.75317 (19)	0.0296 (5)	
C3G	0.38618 (10)	0.38469 (9)	0.74537 (17)	0.0252 (5)	
H3GA	0.3575	0.4087	0.7133	0.030*	
H3GB	0.4079	0.3784	0.7086	0.030*	
C4G	0.42277 (11)	0.40750 (10)	0.83625 (18)	0.0299 (5)	
H4GA	0.4012	0.4125	0.8736	0.036*	
H4GB	0.4520	0.3838	0.8673	0.036*	
C5G	0.44749 (10)	0.45741 (10)	0.82527 (17)	0.0278 (5)	
C6G	0.50172 (11)	0.46090 (10)	0.8309 (2)	0.0328 (6)	
H6GA	0.5230	0.4316	0.8411	0.039*	
C7G	0.52452 (11)	0.50702 (11)	0.8218 (2)	0.0344 (6)	
H7GA	0.5608	0.5084	0.8260	0.041*	
C8G	0.49317 (10)	0.55141 (10)	0.80616 (18)	0.0285 (5)	
C9G	0.43931 (10)	0.54852 (10)	0.80019 (17)	0.0271 (5)	
H9GA	0.4181	0.5778	0.7898	0.033*	
C10G	0.41675 (10)	0.50225 (10)	0.80960 (17)	0.0277 (5)	
H10G	0.3805	0.5010	0.8054	0.033*	
C11G	0.28428 (10)	0.37427 (10)	0.7994 (2)	0.0305 (5)	
H11M	0.2740	0.3969	0.7476	0.037*	
H11N	0.3072	0.3932	0.8523	0.037*	
C12G	0.23253 (11)	0.35676 (11)	0.8134 (2)	0.0334 (6)	
H12M	0.2419	0.3294	0.8574	0.040*	
H12N	0.2182	0.3847	0.8379	0.040*	
C13G	0.18836 (12)	0.33856 (12)	0.7260 (2)	0.0391 (6)	
H13S	0.1578	0.3257	0.7387	0.059*	
H13T	0.2029	0.3121	0.6999	0.059*	
H13U	0.1765	0.3664	0.6843	0.059*	
C1H	0.22601 (11)	0.11376 (10)	0.50764 (19)	0.0315 (5)	
C2H	0.27222 (10)	0.18449 (10)	0.51040 (18)	0.0283 (5)	
C3H	0.17196 (10)	0.19709 (11)	0.47759 (17)	0.0296 (5)	
H3HA	0.1412	0.1786	0.4349	0.036*	
H3HB	0.1768	0.2279	0.4480	0.036*	
C4H	0.15837 (11)	0.21129 (11)	0.55928 (18)	0.0310 (5)	
H4HA	0.1580	0.1810	0.5937	0.037*	
H4HB	0.1863	0.2340	0.5982	0.037*	
C5H	0.10280 (10)	0.23704 (11)	0.52875 (17)	0.0294 (5)	
C6H	0.09705 (11)	0.28935 (11)	0.51339 (19)	0.0316 (5)	
H6HA	0.1279	0.3089	0.5210	0.038*	
C7H	0.04658 (11)	0.31242 (10)	0.4872 (2)	0.0320 (5)	
H7HA	0.0438	0.3474	0.4789	0.038*	
C8H	−0.00050 (11)	0.28369 (10)	0.47299 (18)	0.0290 (5)	
C9H	0.00407 (11)	0.23140 (11)	0.48316 (18)	0.0303 (5)	
H9HA	−0.0272	0.2116	0.4714	0.036*	
C10H	0.05534 (11)	0.20868 (10)	0.51091 (19)	0.0314 (5)	
H10H	0.0580	0.1736	0.5177	0.038*	
C11H	0.17920 (13)	0.08007 (12)	0.5019 (2)	0.0434 (7)	
H11O	0.1465	0.0917	0.4528	0.052*	
H11P	0.1725	0.0829	0.5577	0.052*	
C12H	0.18877 (15)	0.02474 (13)	0.4862 (3)	0.0473 (7)	
H12O	0.2230	0.0140	0.5328	0.057*	
H12P	0.1596	0.0047	0.4930	0.057*	
C13H	0.19112 (16)	0.01352 (16)	0.3966 (3)	0.0597 (10)	
H13V	0.1939	−0.0225	0.3901	0.089*	
H13W	0.2226	0.0299	0.3918	0.089*	
H13X	0.1584	0.0259	0.3498	0.089*	

### Atomic displacement parameters (Å^2^)

**Table:**

	*U*^11^	*U*^22^	*U*^33^	*U*^12^	*U*^13^	*U*^23^
O1A	0.0279 (9)	0.0222 (9)	0.0468 (10)	0.0003 (7)	0.0174 (8)	−0.0001 (8)
O2A	0.0423 (11)	0.0212 (9)	0.0605 (13)	−0.0047 (8)	0.0312 (10)	−0.0036 (9)
O1B	0.0267 (9)	0.0218 (8)	0.0504 (11)	−0.0010 (7)	0.0185 (8)	−0.0004 (8)
O2B	0.0281 (9)	0.0277 (9)	0.0563 (12)	0.0030 (7)	0.0224 (9)	0.0008 (9)
O1C	0.0271 (9)	0.0251 (9)	0.0516 (11)	0.0023 (7)	0.0205 (8)	0.0016 (8)
O2C	0.0235 (8)	0.0275 (9)	0.0479 (11)	−0.0015 (7)	0.0176 (8)	−0.0060 (8)
O1D	0.0270 (9)	0.0228 (9)	0.0530 (11)	0.0002 (7)	0.0212 (8)	−0.0002 (8)
O2D	0.0290 (10)	0.0210 (9)	0.0707 (14)	−0.0028 (8)	0.0212 (9)	0.0001 (9)
O1E	0.0258 (9)	0.0239 (9)	0.0453 (10)	0.0009 (7)	0.0183 (7)	−0.0010 (8)
O2E	0.0351 (10)	0.0254 (9)	0.0530 (12)	−0.0022 (8)	0.0235 (9)	−0.0006 (9)
O1F	0.0269 (9)	0.0222 (9)	0.0490 (11)	0.0006 (7)	0.0151 (8)	−0.0013 (8)
O2F	0.0277 (9)	0.0235 (9)	0.0710 (14)	0.0005 (7)	0.0222 (9)	0.0004 (9)
O1G	0.0288 (9)	0.0245 (9)	0.0576 (12)	0.0018 (7)	0.0232 (9)	−0.0011 (8)
O2G	0.0287 (9)	0.0242 (9)	0.0601 (12)	0.0006 (7)	0.0236 (9)	0.0042 (9)
O1H	0.0295 (9)	0.0233 (9)	0.0602 (13)	0.0009 (7)	0.0216 (9)	0.0031 (9)
O2H	0.0306 (10)	0.0342 (10)	0.0615 (13)	0.0061 (8)	0.0247 (9)	0.0107 (9)
N1A	0.0261 (10)	0.0227 (10)	0.0374 (11)	−0.0015 (8)	0.0108 (9)	−0.0042 (9)
N2A	0.0274 (10)	0.0192 (10)	0.0409 (12)	−0.0023 (8)	0.0140 (9)	−0.0007 (9)
N3A	0.0258 (10)	0.0184 (10)	0.0312 (10)	−0.0024 (8)	0.0096 (8)	−0.0001 (8)
N1B	0.0276 (11)	0.0251 (11)	0.0439 (12)	−0.0029 (9)	0.0175 (9)	−0.0003 (9)
N2B	0.0250 (10)	0.0267 (11)	0.0496 (13)	−0.0019 (9)	0.0203 (10)	−0.0015 (10)
N3B	0.0241 (10)	0.0239 (10)	0.0371 (11)	0.0016 (8)	0.0149 (8)	0.0033 (9)
N1C	0.0237 (10)	0.0246 (11)	0.0343 (11)	0.0009 (8)	0.0098 (8)	0.0037 (9)
N2C	0.0205 (9)	0.0260 (11)	0.0392 (11)	0.0023 (8)	0.0140 (8)	0.0038 (9)
N3C	0.0210 (9)	0.0246 (10)	0.0299 (10)	0.0002 (8)	0.0102 (8)	0.0004 (8)
N1D	0.0269 (10)	0.0224 (10)	0.0433 (12)	−0.0001 (8)	0.0175 (9)	−0.0002 (9)
N2D	0.0288 (10)	0.0170 (10)	0.0483 (13)	0.0007 (8)	0.0188 (9)	−0.0001 (9)
N3D	0.0269 (10)	0.0191 (10)	0.0337 (11)	−0.0022 (8)	0.0134 (8)	0.0004 (8)
N1E	0.0266 (10)	0.0252 (11)	0.0484 (13)	0.0029 (9)	0.0188 (9)	−0.0004 (10)
N2E	0.0302 (11)	0.0222 (10)	0.0453 (12)	0.0003 (8)	0.0198 (9)	−0.0014 (9)
N3E	0.0257 (10)	0.0228 (10)	0.0341 (10)	0.0014 (8)	0.0135 (8)	0.0008 (9)
N1F	0.0279 (11)	0.0230 (11)	0.0522 (14)	0.0002 (9)	0.0177 (10)	0.0022 (10)
N2F	0.0225 (10)	0.0240 (11)	0.0545 (14)	0.0005 (8)	0.0154 (9)	0.0016 (10)
N3F	0.0239 (10)	0.0242 (10)	0.0340 (11)	−0.0007 (8)	0.0106 (8)	−0.0002 (8)
N1G	0.0250 (10)	0.0253 (11)	0.0508 (13)	0.0029 (8)	0.0205 (9)	−0.0012 (10)
N2G	0.0290 (11)	0.0185 (10)	0.0599 (15)	0.0032 (8)	0.0233 (11)	0.0020 (10)
N3G	0.0238 (10)	0.0209 (10)	0.0350 (11)	−0.0006 (8)	0.0124 (8)	−0.0003 (8)
N1H	0.0328 (12)	0.0250 (11)	0.0457 (13)	−0.0009 (9)	0.0201 (10)	0.0032 (10)
N2H	0.0201 (9)	0.0263 (11)	0.0394 (12)	0.0010 (8)	0.0123 (8)	0.0016 (9)
N3H	0.0244 (10)	0.0240 (10)	0.0382 (11)	0.0013 (8)	0.0135 (8)	0.0000 (9)
C1A	0.0241 (11)	0.0268 (12)	0.0294 (12)	−0.0035 (9)	0.0081 (9)	−0.0020 (10)
C2A	0.0293 (12)	0.0202 (11)	0.0293 (12)	−0.0028 (9)	0.0104 (9)	−0.0015 (9)
C3A	0.0271 (11)	0.0213 (11)	0.0318 (12)	−0.0042 (9)	0.0130 (9)	−0.0004 (10)
C4A	0.0320 (12)	0.0225 (12)	0.0309 (12)	−0.0055 (10)	0.0123 (10)	0.0007 (10)
C5A	0.0268 (11)	0.0214 (12)	0.0284 (11)	−0.0035 (9)	0.0094 (9)	−0.0010 (9)
C6A	0.0290 (12)	0.0213 (12)	0.0429 (14)	0.0033 (10)	0.0155 (11)	−0.0019 (11)
C7A	0.0276 (12)	0.0294 (14)	0.0437 (14)	−0.0003 (10)	0.0205 (11)	−0.0001 (11)
C8A	0.0284 (12)	0.0216 (12)	0.0342 (12)	−0.0034 (9)	0.0130 (10)	−0.0011 (10)
C9A	0.0276 (12)	0.0196 (11)	0.0387 (13)	0.0000 (9)	0.0141 (10)	−0.0022 (10)
C10A	0.0227 (11)	0.0285 (13)	0.0350 (13)	−0.0006 (10)	0.0131 (9)	−0.0003 (10)
C11A	0.0303 (13)	0.0226 (12)	0.0390 (13)	0.0004 (10)	0.0096 (10)	−0.0013 (10)
C12A	0.0277 (13)	0.0348 (15)	0.0544 (17)	−0.0031 (11)	0.0150 (12)	−0.0097 (13)
C13A	0.0320 (15)	0.0474 (18)	0.069 (2)	0.0048 (13)	0.0190 (14)	−0.0090 (16)
C1B	0.0271 (12)	0.0259 (13)	0.0344 (13)	0.0029 (10)	0.0123 (10)	0.0011 (10)
C2B	0.0230 (12)	0.0285 (13)	0.0356 (13)	0.0026 (10)	0.0121 (10)	0.0018 (11)
C3B	0.0257 (11)	0.0282 (13)	0.0309 (12)	0.0018 (10)	0.0133 (9)	−0.0007 (10)
C4B	0.0316 (13)	0.0346 (14)	0.0330 (13)	0.0080 (11)	0.0142 (10)	0.0017 (11)
C5B	0.0257 (12)	0.0321 (13)	0.0298 (12)	0.0052 (10)	0.0102 (9)	0.0016 (10)
C6B	0.0248 (11)	0.0304 (13)	0.0316 (12)	−0.0014 (10)	0.0118 (9)	0.0024 (10)
C7B	0.0279 (12)	0.0233 (12)	0.0329 (12)	−0.0030 (10)	0.0096 (9)	0.0004 (10)
C8B	0.0236 (11)	0.0290 (13)	0.0301 (12)	0.0032 (10)	0.0100 (9)	−0.0002 (10)
C9B	0.0222 (11)	0.0262 (13)	0.0378 (13)	−0.0022 (9)	0.0131 (10)	0.0006 (10)
C10B	0.0262 (12)	0.0240 (12)	0.0366 (13)	0.0030 (10)	0.0103 (10)	−0.0009 (10)
C11B	0.0255 (12)	0.0255 (13)	0.0456 (15)	0.0007 (10)	0.0159 (11)	0.0040 (11)
C12B	0.0307 (13)	0.0241 (13)	0.0496 (16)	0.0007 (10)	0.0180 (11)	0.0011 (11)
C13B	0.0468 (17)	0.0261 (14)	0.086 (3)	−0.0051 (13)	0.0314 (17)	0.0012 (16)
C1C	0.0237 (11)	0.0260 (12)	0.0267 (11)	0.0035 (9)	0.0080 (9)	0.0032 (10)
C2C	0.0203 (11)	0.0288 (13)	0.0311 (12)	0.0025 (9)	0.0101 (9)	0.0026 (10)
C3C	0.0197 (11)	0.0281 (12)	0.0309 (12)	0.0010 (9)	0.0110 (9)	−0.0037 (10)
C4C	0.0231 (11)	0.0300 (13)	0.0303 (12)	0.0034 (10)	0.0100 (9)	−0.0026 (10)
C5C	0.0207 (11)	0.0318 (13)	0.0267 (11)	0.0013 (9)	0.0084 (9)	−0.0015 (10)
C6C	0.0213 (11)	0.0284 (13)	0.0375 (13)	−0.0042 (9)	0.0123 (10)	−0.0006 (11)
C7C	0.0270 (12)	0.0220 (12)	0.0362 (13)	−0.0037 (9)	0.0121 (10)	−0.0054 (10)
C8C	0.0227 (11)	0.0241 (12)	0.0289 (12)	0.0028 (9)	0.0102 (9)	0.0022 (10)
C9C	0.0199 (11)	0.0238 (12)	0.0321 (12)	−0.0026 (9)	0.0088 (9)	0.0027 (9)
C10C	0.0256 (11)	0.0210 (11)	0.0309 (12)	0.0006 (9)	0.0093 (9)	0.0012 (9)
C11C	0.0246 (11)	0.0292 (13)	0.0372 (13)	0.0004 (10)	0.0145 (10)	0.0031 (11)
C12C	0.0230 (12)	0.0277 (13)	0.0496 (15)	0.0023 (10)	0.0131 (11)	0.0041 (12)
C13C	0.0389 (16)	0.0282 (14)	0.085 (3)	−0.0022 (12)	0.0261 (16)	0.0058 (15)
C1D	0.0227 (11)	0.0237 (12)	0.0291 (12)	−0.0007 (9)	0.0108 (9)	0.0001 (9)
C2D	0.0273 (12)	0.0189 (11)	0.0383 (13)	−0.0016 (9)	0.0139 (10)	−0.0006 (10)
C3D	0.0276 (12)	0.0190 (11)	0.0307 (12)	−0.0044 (9)	0.0107 (9)	−0.0005 (9)
C4D	0.0310 (12)	0.0241 (12)	0.0295 (12)	−0.0025 (10)	0.0099 (9)	0.0008 (10)
C5D	0.0305 (12)	0.0249 (12)	0.0281 (12)	−0.0039 (10)	0.0121 (9)	0.0022 (10)
C6D	0.0304 (13)	0.0211 (12)	0.0386 (13)	0.0026 (10)	0.0126 (10)	0.0025 (10)
C7D	0.0260 (12)	0.0254 (13)	0.0416 (14)	0.0016 (10)	0.0121 (10)	0.0017 (11)
C8D	0.0333 (13)	0.0215 (12)	0.0359 (13)	−0.0017 (10)	0.0159 (10)	0.0008 (10)
C9D	0.0295 (12)	0.0220 (12)	0.0371 (13)	0.0021 (10)	0.0164 (10)	0.0035 (10)
C10D	0.0245 (11)	0.0278 (12)	0.0350 (12)	−0.0008 (9)	0.0135 (9)	0.0018 (10)
C11D	0.0269 (12)	0.0230 (12)	0.0343 (13)	−0.0002 (9)	0.0128 (10)	−0.0001 (10)
C12D	0.0314 (13)	0.0248 (12)	0.0418 (14)	0.0009 (10)	0.0181 (11)	0.0040 (11)
C13D	0.0322 (14)	0.0369 (15)	0.0546 (17)	0.0084 (12)	0.0206 (13)	0.0033 (13)
C1E	0.0271 (12)	0.0267 (13)	0.0361 (13)	−0.0003 (10)	0.0129 (10)	−0.0022 (10)
C2E	0.0281 (12)	0.0222 (12)	0.0321 (12)	−0.0004 (9)	0.0123 (10)	−0.0023 (10)
C3E	0.0284 (12)	0.0221 (12)	0.0346 (12)	0.0000 (9)	0.0149 (10)	0.0000 (10)
C4E	0.0390 (14)	0.0307 (13)	0.0295 (12)	−0.0040 (11)	0.0121 (10)	−0.0016 (10)
C5E	0.0318 (12)	0.0264 (12)	0.0302 (12)	−0.0038 (10)	0.0107 (10)	−0.0051 (10)
C6E	0.0312 (13)	0.0242 (12)	0.0384 (14)	0.0028 (10)	0.0079 (10)	−0.0035 (11)
C7E	0.0246 (12)	0.0298 (14)	0.0421 (14)	−0.0006 (10)	0.0130 (10)	−0.0066 (11)
C8E	0.0299 (12)	0.0239 (12)	0.0332 (12)	−0.0052 (10)	0.0140 (10)	−0.0051 (10)
C9E	0.0285 (12)	0.0245 (12)	0.0371 (13)	0.0030 (10)	0.0140 (10)	−0.0031 (10)
C10E	0.0261 (12)	0.0303 (13)	0.0318 (12)	−0.0024 (10)	0.0122 (10)	−0.0047 (10)
C11E	0.0321 (14)	0.0285 (14)	0.0539 (17)	0.0051 (11)	0.0191 (12)	−0.0024 (12)
C12E	0.0464 (19)	0.0421 (19)	0.082 (3)	0.0072 (15)	0.0268 (18)	−0.0044 (18)
C13E	0.0341 (17)	0.067 (2)	0.083 (3)	0.0009 (16)	0.0108 (17)	−0.011 (2)
C1F	0.0285 (12)	0.0249 (12)	0.0334 (12)	−0.0010 (10)	0.0126 (10)	−0.0010 (10)
C2F	0.0233 (11)	0.0268 (13)	0.0335 (12)	0.0001 (9)	0.0114 (10)	−0.0018 (10)
C3F	0.0224 (11)	0.0267 (12)	0.0331 (12)	0.0022 (9)	0.0101 (9)	0.0025 (10)
C4F	0.0268 (12)	0.0379 (14)	0.0339 (13)	0.0055 (11)	0.0102 (10)	−0.0012 (11)
C5F	0.0266 (12)	0.0353 (14)	0.0313 (12)	0.0047 (10)	0.0126 (10)	−0.0012 (11)
C6F	0.0269 (13)	0.0324 (15)	0.0565 (17)	−0.0047 (11)	0.0168 (12)	−0.0025 (13)
C7F	0.0327 (14)	0.0243 (13)	0.0641 (19)	−0.0029 (11)	0.0221 (13)	−0.0014 (13)
C8F	0.0297 (13)	0.0272 (13)	0.0407 (14)	0.0006 (10)	0.0173 (11)	−0.0002 (11)
C9F	0.0284 (12)	0.0253 (12)	0.0423 (14)	−0.0040 (10)	0.0186 (11)	−0.0004 (11)
C10F	0.0338 (13)	0.0238 (12)	0.0383 (13)	0.0038 (10)	0.0170 (11)	0.0024 (11)
C11F	0.0275 (12)	0.0300 (14)	0.0437 (14)	−0.0033 (10)	0.0167 (11)	0.0016 (11)
C12F	0.0335 (14)	0.0281 (14)	0.0489 (16)	−0.0056 (11)	0.0094 (12)	0.0099 (12)
C13F	0.0344 (15)	0.0290 (14)	0.0601 (19)	0.0035 (12)	0.0060 (13)	0.0002 (14)
C1G	0.0237 (11)	0.0242 (12)	0.0350 (13)	−0.0011 (9)	0.0116 (9)	−0.0018 (10)
C2G	0.0253 (12)	0.0254 (12)	0.0386 (13)	−0.0015 (10)	0.0125 (10)	−0.0010 (10)
C3G	0.0243 (11)	0.0197 (11)	0.0337 (12)	−0.0036 (9)	0.0131 (9)	0.0006 (9)
C4G	0.0289 (12)	0.0266 (12)	0.0324 (13)	−0.0034 (10)	0.0093 (10)	0.0026 (10)
C5G	0.0288 (12)	0.0240 (12)	0.0289 (12)	−0.0049 (10)	0.0087 (9)	−0.0010 (9)
C6G	0.0272 (12)	0.0243 (12)	0.0482 (15)	0.0044 (10)	0.0156 (11)	0.0018 (11)
C7G	0.0258 (12)	0.0284 (13)	0.0516 (16)	0.0010 (10)	0.0172 (11)	0.0034 (12)
C8G	0.0284 (12)	0.0233 (12)	0.0363 (13)	−0.0021 (10)	0.0148 (10)	−0.0022 (10)
C9G	0.0245 (12)	0.0227 (12)	0.0338 (12)	0.0006 (9)	0.0104 (10)	−0.0018 (10)
C10G	0.0234 (11)	0.0281 (13)	0.0331 (12)	−0.0007 (9)	0.0122 (9)	−0.0028 (10)
C11G	0.0273 (12)	0.0224 (12)	0.0437 (14)	0.0010 (10)	0.0156 (10)	−0.0042 (11)
C12G	0.0322 (13)	0.0322 (14)	0.0433 (14)	0.0035 (11)	0.0226 (11)	−0.0019 (11)
C13G	0.0297 (13)	0.0367 (15)	0.0541 (17)	0.0001 (11)	0.0193 (12)	−0.0026 (13)
C1H	0.0338 (13)	0.0245 (13)	0.0411 (14)	−0.0005 (11)	0.0197 (11)	−0.0004 (11)
C2H	0.0263 (12)	0.0240 (13)	0.0354 (13)	0.0004 (9)	0.0122 (10)	0.0007 (10)
C3H	0.0252 (12)	0.0333 (14)	0.0296 (12)	0.0037 (10)	0.0094 (9)	0.0040 (10)
C4H	0.0290 (12)	0.0310 (13)	0.0324 (13)	0.0044 (10)	0.0105 (10)	0.0028 (11)
C5H	0.0275 (12)	0.0339 (14)	0.0303 (12)	0.0027 (10)	0.0148 (10)	0.0001 (10)
C6H	0.0263 (12)	0.0297 (13)	0.0407 (14)	−0.0043 (10)	0.0146 (10)	−0.0021 (11)
C7H	0.0349 (13)	0.0236 (12)	0.0437 (14)	0.0005 (10)	0.0220 (11)	0.0016 (11)
C8H	0.0290 (12)	0.0295 (13)	0.0330 (12)	0.0035 (10)	0.0167 (10)	0.0012 (10)
C9H	0.0272 (12)	0.0293 (13)	0.0383 (13)	−0.0033 (10)	0.0167 (10)	0.0002 (11)
C10H	0.0331 (13)	0.0238 (12)	0.0417 (14)	0.0012 (10)	0.0189 (11)	−0.0005 (11)
C11H	0.0416 (16)	0.0343 (15)	0.065 (2)	−0.0104 (13)	0.0319 (15)	−0.0061 (14)
C12H	0.0490 (17)	0.0348 (16)	0.060 (2)	−0.0111 (14)	0.0229 (15)	−0.0029 (15)
C13H	0.056 (2)	0.058 (2)	0.072 (2)	−0.0199 (18)	0.0317 (18)	−0.0254 (19)

### Geometric parameters (Å, º)

**Table:**

O1A—C2A	1.246 (3)	C7C—H7CA	0.9300
O2A—C8A	1.364 (3)	C8C—C9C	1.394 (4)
O2A—H2A	0.8200	C9C—C10C	1.390 (4)
O1B—C2B	1.245 (3)	C9C—H9CA	0.9300
O2B—C8B	1.364 (3)	C10C—H10C	0.9300
O2B—H2B	0.8200	C11C—C12C	1.517 (4)
O1C—C2C	1.254 (3)	C11C—H11E	0.9700
O2C—C8C	1.365 (3)	C11C—H11F	0.9700
O2C—H2C	0.8200	C12C—C13C	1.525 (4)
O1D—C2D	1.255 (3)	C12C—H12E	0.9700
O2D—C8D	1.364 (3)	C12C—H12F	0.9700
O2D—H2D	0.8200	C13C—H13G	0.9600
O1E—C2E	1.251 (3)	C13C—H13H	0.9600
O2E—C8E	1.358 (3)	C13C—H13I	0.9600
O2E—H2E	0.8200	C1D—C11D	1.489 (3)
O1F—C2F	1.256 (3)	C3D—C4D	1.518 (3)
O2F—C8F	1.354 (3)	C3D—H3DA	0.9700
O2F—H2F	0.8200	C3D—H3DB	0.9700
O1G—C2G	1.249 (3)	C4D—C5D	1.510 (3)
O2G—C8G	1.356 (3)	C4D—H4DA	0.9700
O2G—H2G	0.8200	C4D—H4DB	0.9700
O1H—C2H	1.252 (3)	C5D—C10D	1.386 (4)
O2H—C8H	1.369 (3)	C5D—C6D	1.401 (4)
O2H—H2H	0.8200	C6D—C7D	1.389 (4)
N1A—C1A	1.295 (3)	C6D—H6DA	0.9300
N1A—N2A	1.383 (3)	C7D—C8D	1.392 (4)
N2A—C2A	1.342 (3)	C7D—H7DA	0.9300
N2A—H2AA	0.8600	C8D—C9D	1.389 (4)
N3A—C2A	1.373 (3)	C9D—C10D	1.386 (4)
N3A—C1A	1.387 (3)	C9D—H9DA	0.9300
N3A—C3A	1.459 (3)	C10D—H10D	0.9300
N1B—C1B	1.302 (3)	C11D—C12D	1.521 (4)
N1B—N2B	1.379 (3)	C11D—H11G	0.9700
N2B—C2B	1.341 (3)	C11D—H11H	0.9700
N2B—H2BA	0.8600	C12D—C13D	1.522 (4)
N3B—C1B	1.376 (3)	C12D—H12G	0.9700
N3B—C2B	1.378 (3)	C12D—H12H	0.9700
N3B—C3B	1.464 (3)	C13D—H13J	0.9600
N1C—C1C	1.291 (3)	C13D—H13K	0.9600
N1C—N2C	1.381 (3)	C13D—H13L	0.9600
N2C—C2C	1.339 (3)	C1E—C11E	1.503 (4)
N2C—H2CA	0.8600	C3E—C4E	1.519 (4)
N3C—C2C	1.375 (3)	C3E—H3EA	0.9700
N3C—C1C	1.381 (3)	C3E—H3EB	0.9700
N3C—C3C	1.469 (3)	C4E—C5E	1.516 (4)
N1D—C1D	1.302 (3)	C4E—H4EA	0.9700
N1D—N2D	1.384 (3)	C4E—H4EB	0.9700
N2D—C2D	1.333 (3)	C5E—C10E	1.389 (4)
N2D—H2DA	0.8600	C5E—C6E	1.399 (4)
N3D—C2D	1.375 (3)	C6E—C7E	1.378 (4)
N3D—C1D	1.383 (3)	C6E—H6EA	0.9300
N3D—C3D	1.460 (3)	C7E—C8E	1.405 (4)
N1E—C1E	1.303 (3)	C7E—H7EA	0.9300
N1E—N2E	1.374 (3)	C8E—C9E	1.395 (4)
N2E—C2E	1.344 (3)	C9E—C10E	1.386 (4)
N2E—H2EA	0.8600	C9E—H9EA	0.9300
N3E—C2E	1.371 (3)	C10E—H10E	0.9300
N3E—C1E	1.382 (3)	C11E—C12E	1.529 (5)
N3E—C3E	1.477 (3)	C11E—H11I	0.9700
N1F—C1F	1.289 (3)	C11E—H11J	0.9700
N1F—N2F	1.389 (3)	C12E—C13E	1.525 (6)
N2F—C2F	1.338 (3)	C12E—H12I	0.9700
N2F—H2FA	0.8600	C12E—H12J	0.9700
N3F—C2F	1.369 (3)	C13E—H13M	0.9600
N3F—C1F	1.380 (3)	C13E—H13N	0.9600
N3F—C3F	1.463 (3)	C13E—H13O	0.9600
N1G—C1G	1.295 (3)	C1F—C11F	1.498 (4)
N1G—N2G	1.386 (3)	C3F—C4F	1.522 (4)
N2G—C2G	1.334 (3)	C3F—H3FA	0.9700
N2G—H2GA	0.8600	C3F—H3FB	0.9700
N3G—C2G	1.376 (3)	C4F—C5F	1.515 (3)
N3G—C1G	1.386 (3)	C4F—H4FA	0.9700
N3G—C3G	1.467 (3)	C4F—H4FB	0.9700
N1H—C1H	1.297 (4)	C5F—C6F	1.384 (4)
N1H—N2H	1.375 (3)	C5F—C10F	1.390 (4)
N2H—C2H	1.347 (3)	C6F—C7F	1.394 (4)
N2H—H2HA	0.8600	C6F—H6FA	0.9300
N3H—C2H	1.369 (3)	C7F—C8F	1.387 (4)
N3H—C1H	1.379 (3)	C7F—H7FA	0.9300
N3H—C3H	1.468 (3)	C8F—C9F	1.392 (4)
C1A—C11A	1.491 (4)	C9F—C10F	1.391 (4)
C3A—C4A	1.525 (3)	C9F—H9FA	0.9300
C3A—H3AA	0.9700	C10F—H10F	0.9300
C3A—H3AB	0.9700	C11F—C12F	1.528 (4)
C4A—C5A	1.512 (3)	C11F—H11K	0.9700
C4A—H4AA	0.9700	C11F—H11L	0.9700
C4A—H4AB	0.9700	C12F—C13F	1.510 (5)
C5A—C6A	1.399 (4)	C12F—H12K	0.9700
C5A—C10A	1.402 (3)	C12F—H12L	0.9700
C6A—C7A	1.386 (4)	C13F—H13P	0.9600
C6A—H6AA	0.9300	C13F—H13Q	0.9600
C7A—C8A	1.387 (4)	C13F—H13R	0.9600
C7A—H7AA	0.9300	C1G—C11G	1.498 (3)
C8A—C9A	1.397 (4)	C3G—C4G	1.529 (4)
C9A—C10A	1.379 (4)	C3G—H3GA	0.9700
C9A—H9AA	0.9300	C3G—H3GB	0.9700
C10A—H10A	0.9300	C4G—C5G	1.505 (3)
C11A—C12A	1.523 (4)	C4G—H4GA	0.9700
C11A—H11A	0.9700	C4G—H4GB	0.9700
C11A—H11B	0.9700	C5G—C6G	1.396 (4)
C12A—C13A	1.532 (4)	C5G—C10G	1.400 (4)
C12A—H12A	0.9700	C6G—C7G	1.386 (4)
C12A—H12B	0.9700	C6G—H6GA	0.9300
C13A—H13A	0.9600	C7G—C8G	1.398 (4)
C13A—H13B	0.9600	C7G—H7GA	0.9300
C13A—H13C	0.9600	C8G—C9G	1.384 (4)
C1B—C11B	1.496 (4)	C9G—C10G	1.387 (4)
C3B—C4B	1.521 (3)	C9G—H9GA	0.9300
C3B—H3BA	0.9700	C10G—H10G	0.9300
C3B—H3BB	0.9700	C11G—C12G	1.529 (4)
C4B—C5B	1.515 (3)	C11G—H11M	0.9700
C4B—H4BA	0.9700	C11G—H11N	0.9700
C4B—H4BB	0.9700	C12G—C13G	1.519 (4)
C5B—C6B	1.391 (4)	C12G—H12M	0.9700
C5B—C10B	1.395 (4)	C12G—H12N	0.9700
C6B—C7B	1.382 (4)	C13G—H13S	0.9600
C6B—H6BA	0.9300	C13G—H13T	0.9600
C7B—C8B	1.394 (4)	C13G—H13U	0.9600
C7B—H7BA	0.9300	C1H—C11H	1.491 (4)
C8B—C9B	1.392 (4)	C3H—C4H	1.517 (4)
C9B—C10B	1.379 (4)	C3H—H3HA	0.9700
C9B—H9BA	0.9300	C3H—H3HB	0.9700
C10B—H10B	0.9300	C4H—C5H	1.516 (4)
C11B—C12B	1.526 (4)	C4H—H4HA	0.9700
C11B—H11C	0.9700	C4H—H4HB	0.9700
C11B—H11D	0.9700	C5H—C10H	1.389 (4)
C12B—C13B	1.528 (4)	C5H—C6H	1.398 (4)
C12B—H12C	0.9700	C6H—C7H	1.374 (4)
C12B—H12D	0.9700	C6H—H6HA	0.9300
C13B—H13D	0.9600	C7H—C8H	1.394 (4)
C13B—H13E	0.9600	C7H—H7HA	0.9300
C13B—H13F	0.9600	C8H—C9H	1.387 (4)
C1C—C11C	1.495 (4)	C9H—C10H	1.387 (4)
C3C—C4C	1.532 (3)	C9H—H9HA	0.9300
C3C—H3CA	0.9700	C10H—H10H	0.9300
C3C—H3CB	0.9700	C11H—C12H	1.515 (4)
C4C—C5C	1.521 (3)	C11H—H11O	0.9700
C4C—H4CA	0.9700	C11H—H11P	0.9700
C4C—H4CB	0.9700	C12H—C13H	1.478 (5)
C5C—C6C	1.385 (4)	C12H—H12O	0.9700
C5C—C10C	1.396 (3)	C12H—H12P	0.9700
C6C—C7C	1.384 (4)	C13H—H13V	0.9600
C6C—H6CA	0.9300	C13H—H13W	0.9600
C7C—C8C	1.385 (3)	C13H—H13X	0.9600
			
C8A—O2A—H2A	109.5	C7D—C6D—H6DA	119.5
C8B—O2B—H2B	109.5	C5D—C6D—H6DA	119.5
C8C—O2C—H2C	109.5	C6D—C7D—C8D	119.9 (2)
C8D—O2D—H2D	109.5	C6D—C7D—H7DA	120.0
C8E—O2E—H2E	109.5	C8D—C7D—H7DA	120.0
C8F—O2F—H2F	109.5	O2D—C8D—C9D	122.7 (2)
C8G—O2G—H2G	109.5	O2D—C8D—C7D	117.7 (2)
C8H—O2H—H2H	109.5	C9D—C8D—C7D	119.6 (2)
C1A—N1A—N2A	104.5 (2)	C10D—C9D—C8D	119.7 (2)
C2A—N2A—N1A	112.7 (2)	C10D—C9D—H9DA	120.1
C2A—N2A—H2AA	123.6	C8D—C9D—H9DA	120.1
N1A—N2A—H2AA	123.6	C5D—C10D—C9D	121.9 (2)
C2A—N3A—C1A	107.5 (2)	C5D—C10D—H10D	119.1
C2A—N3A—C3A	124.6 (2)	C9D—C10D—H10D	119.1
C1A—N3A—C3A	127.8 (2)	C1D—C11D—C12D	113.0 (2)
C1B—N1B—N2B	103.7 (2)	C1D—C11D—H11G	109.0
C2B—N2B—N1B	113.5 (2)	C12D—C11D—H11G	109.0
C2B—N2B—H2BA	123.2	C1D—C11D—H11H	109.0
N1B—N2B—H2BA	123.2	C12D—C11D—H11H	109.0
C1B—N3B—C2B	107.6 (2)	H11G—C11D—H11H	107.8
C1B—N3B—C3B	128.2 (2)	C11D—C12D—C13D	112.1 (2)
C2B—N3B—C3B	124.2 (2)	C11D—C12D—H12G	109.2
C1C—N1C—N2C	104.2 (2)	C13D—C12D—H12G	109.2
C2C—N2C—N1C	112.7 (2)	C11D—C12D—H12H	109.2
C2C—N2C—H2CA	123.7	C13D—C12D—H12H	109.2
N1C—N2C—H2CA	123.7	H12G—C12D—H12H	107.9
C2C—N3C—C1C	106.9 (2)	C12D—C13D—H13J	109.5
C2C—N3C—C3C	125.3 (2)	C12D—C13D—H13K	109.5
C1C—N3C—C3C	127.8 (2)	H13J—C13D—H13K	109.5
C1D—N1D—N2D	104.1 (2)	C12D—C13D—H13L	109.5
C2D—N2D—N1D	113.1 (2)	H13J—C13D—H13L	109.5
C2D—N2D—H2DA	123.5	H13K—C13D—H13L	109.5
N1D—N2D—H2DA	123.5	N1E—C1E—N3E	111.5 (2)
C2D—N3D—C1D	107.5 (2)	N1E—C1E—C11E	125.2 (2)
C2D—N3D—C3D	124.5 (2)	N3E—C1E—C11E	123.3 (2)
C1D—N3D—C3D	127.7 (2)	O1E—C2E—N2E	128.1 (2)
C1E—N1E—N2E	104.0 (2)	O1E—C2E—N3E	127.8 (2)
C2E—N2E—N1E	113.1 (2)	N2E—C2E—N3E	104.2 (2)
C2E—N2E—H2EA	123.5	N3E—C3E—C4E	114.4 (2)
N1E—N2E—H2EA	123.5	N3E—C3E—H3EA	108.7
C2E—N3E—C1E	107.2 (2)	C4E—C3E—H3EA	108.7
C2E—N3E—C3E	124.1 (2)	N3E—C3E—H3EB	108.7
C1E—N3E—C3E	128.2 (2)	C4E—C3E—H3EB	108.7
C1F—N1F—N2F	104.2 (2)	H3EA—C3E—H3EB	107.6
C2F—N2F—N1F	112.4 (2)	C5E—C4E—C3E	109.6 (2)
C2F—N2F—H2FA	123.8	C5E—C4E—H4EA	109.8
N1F—N2F—H2FA	123.8	C3E—C4E—H4EA	109.8
C2F—N3F—C1F	107.4 (2)	C5E—C4E—H4EB	109.8
C2F—N3F—C3F	124.6 (2)	C3E—C4E—H4EB	109.8
C1F—N3F—C3F	128.0 (2)	H4EA—C4E—H4EB	108.2
C1G—N1G—N2G	104.0 (2)	C10E—C5E—C6E	117.1 (2)
C2G—N2G—N1G	113.1 (2)	C10E—C5E—C4E	121.2 (2)
C2G—N2G—H2GA	123.4	C6E—C5E—C4E	121.7 (2)
N1G—N2G—H2GA	123.4	C7E—C6E—C5E	121.8 (3)
C2G—N3G—C1G	107.3 (2)	C7E—C6E—H6EA	119.1
C2G—N3G—C3G	124.7 (2)	C5E—C6E—H6EA	119.1
C1G—N3G—C3G	127.9 (2)	C6E—C7E—C8E	120.4 (2)
C1H—N1H—N2H	104.6 (2)	C6E—C7E—H7EA	119.8
C2H—N2H—N1H	112.4 (2)	C8E—C7E—H7EA	119.8
C2H—N2H—H2HA	123.8	O2E—C8E—C9E	123.0 (2)
N1H—N2H—H2HA	123.8	O2E—C8E—C7E	118.7 (2)
C2H—N3H—C1H	107.4 (2)	C9E—C8E—C7E	118.3 (2)
C2H—N3H—C3H	124.3 (2)	C10E—C9E—C8E	120.1 (2)
C1H—N3H—C3H	128.1 (2)	C10E—C9E—H9EA	119.9
N1A—C1A—N3A	111.1 (2)	C8E—C9E—H9EA	119.9
N1A—C1A—C11A	125.8 (2)	C9E—C10E—C5E	122.2 (2)
N3A—C1A—C11A	123.0 (2)	C9E—C10E—H10E	118.9
O1A—C2A—N2A	128.6 (2)	C5E—C10E—H10E	118.9
O1A—C2A—N3A	127.3 (2)	C1E—C11E—C12E	112.3 (3)
N2A—C2A—N3A	104.1 (2)	C1E—C11E—H11I	109.1
N3A—C3A—C4A	112.8 (2)	C12E—C11E—H11I	109.1
N3A—C3A—H3AA	109.0	C1E—C11E—H11J	109.1
C4A—C3A—H3AA	109.0	C12E—C11E—H11J	109.1
N3A—C3A—H3AB	109.0	H11I—C11E—H11J	107.9
C4A—C3A—H3AB	109.0	C13E—C12E—C11E	113.5 (3)
H3AA—C3A—H3AB	107.8	C13E—C12E—H12I	108.9
C5A—C4A—C3A	110.8 (2)	C11E—C12E—H12I	108.9
C5A—C4A—H4AA	109.5	C13E—C12E—H12J	108.9
C3A—C4A—H4AA	109.5	C11E—C12E—H12J	108.9
C5A—C4A—H4AB	109.5	H12I—C12E—H12J	107.7
C3A—C4A—H4AB	109.5	C12E—C13E—H13M	109.5
H4AA—C4A—H4AB	108.1	C12E—C13E—H13N	109.5
C6A—C5A—C10A	117.5 (2)	H13M—C13E—H13N	109.5
C6A—C5A—C4A	121.5 (2)	C12E—C13E—H13O	109.5
C10A—C5A—C4A	121.0 (2)	H13M—C13E—H13O	109.5
C7A—C6A—C5A	121.3 (2)	H13N—C13E—H13O	109.5
C7A—C6A—H6AA	119.4	N1F—C1F—N3F	111.6 (2)
C5A—C6A—H6AA	119.4	N1F—C1F—C11F	124.5 (2)
C6A—C7A—C8A	120.4 (2)	N3F—C1F—C11F	123.9 (2)
C6A—C7A—H7AA	119.8	O1F—C2F—N2F	127.5 (2)
C8A—C7A—H7AA	119.8	O1F—C2F—N3F	128.2 (2)
O2A—C8A—C7A	117.7 (2)	N2F—C2F—N3F	104.4 (2)
O2A—C8A—C9A	123.1 (2)	N3F—C3F—C4F	111.6 (2)
C7A—C8A—C9A	119.3 (2)	N3F—C3F—H3FA	109.3
C10A—C9A—C8A	120.1 (2)	C4F—C3F—H3FA	109.3
C10A—C9A—H9AA	120.0	N3F—C3F—H3FB	109.3
C8A—C9A—H9AA	120.0	C4F—C3F—H3FB	109.3
C9A—C10A—C5A	121.5 (2)	H3FA—C3F—H3FB	108.0
C9A—C10A—H10A	119.2	C5F—C4F—C3F	112.2 (2)
C5A—C10A—H10A	119.2	C5F—C4F—H4FA	109.2
C1A—C11A—C12A	113.2 (2)	C3F—C4F—H4FA	109.2
C1A—C11A—H11A	108.9	C5F—C4F—H4FB	109.2
C12A—C11A—H11A	108.9	C3F—C4F—H4FB	109.2
C1A—C11A—H11B	108.9	H4FA—C4F—H4FB	107.9
C12A—C11A—H11B	108.9	C6F—C5F—C10F	117.8 (2)
H11A—C11A—H11B	107.8	C6F—C5F—C4F	121.8 (3)
C11A—C12A—C13A	110.5 (3)	C10F—C5F—C4F	120.5 (3)
C11A—C12A—H12A	109.5	C5F—C6F—C7F	121.4 (3)
C13A—C12A—H12A	109.5	C5F—C6F—H6FA	119.3
C11A—C12A—H12B	109.5	C7F—C6F—H6FA	119.3
C13A—C12A—H12B	109.5	C8F—C7F—C6F	120.2 (3)
H12A—C12A—H12B	108.1	C8F—C7F—H7FA	119.9
C12A—C13A—H13A	109.5	C6F—C7F—H7FA	119.9
C12A—C13A—H13B	109.5	O2F—C8F—C7F	117.6 (2)
H13A—C13A—H13B	109.5	O2F—C8F—C9F	123.1 (2)
C12A—C13A—H13C	109.5	C7F—C8F—C9F	119.3 (2)
H13A—C13A—H13C	109.5	C10F—C9F—C8F	119.6 (2)
H13B—C13A—H13C	109.5	C10F—C9F—H9FA	120.2
N1B—C1B—N3B	111.6 (2)	C8F—C9F—H9FA	120.2
N1B—C1B—C11B	124.6 (2)	C5F—C10F—C9F	121.8 (2)
N3B—C1B—C11B	123.8 (2)	C5F—C10F—H10F	119.1
O1B—C2B—N2B	128.8 (2)	C9F—C10F—H10F	119.1
O1B—C2B—N3B	127.6 (2)	C1F—C11F—C12F	112.6 (2)
N2B—C2B—N3B	103.6 (2)	C1F—C11F—H11K	109.1
N3B—C3B—C4B	111.7 (2)	C12F—C11F—H11K	109.1
N3B—C3B—H3BA	109.3	C1F—C11F—H11L	109.1
C4B—C3B—H3BA	109.3	C12F—C11F—H11L	109.1
N3B—C3B—H3BB	109.3	H11K—C11F—H11L	107.8
C4B—C3B—H3BB	109.3	C13F—C12F—C11F	113.1 (3)
H3BA—C3B—H3BB	107.9	C13F—C12F—H12K	109.0
C5B—C4B—C3B	112.2 (2)	C11F—C12F—H12K	109.0
C5B—C4B—H4BA	109.2	C13F—C12F—H12L	109.0
C3B—C4B—H4BA	109.2	C11F—C12F—H12L	109.0
C5B—C4B—H4BB	109.2	H12K—C12F—H12L	107.8
C3B—C4B—H4BB	109.2	C12F—C13F—H13P	109.5
H4BA—C4B—H4BB	107.9	C12F—C13F—H13Q	109.5
C6B—C5B—C10B	117.9 (2)	H13P—C13F—H13Q	109.5
C6B—C5B—C4B	121.6 (2)	C12F—C13F—H13R	109.5
C10B—C5B—C4B	120.6 (2)	H13P—C13F—H13R	109.5
C7B—C6B—C5B	121.0 (2)	H13Q—C13F—H13R	109.5
C7B—C6B—H6BA	119.5	N1G—C1G—N3G	111.4 (2)
C5B—C6B—H6BA	119.5	N1G—C1G—C11G	125.2 (2)
C6B—C7B—C8B	120.4 (2)	N3G—C1G—C11G	123.4 (2)
C6B—C7B—H7BA	119.8	O1G—C2G—N2G	128.8 (3)
C8B—C7B—H7BA	119.8	O1G—C2G—N3G	127.1 (2)
O2B—C8B—C9B	122.6 (2)	N2G—C2G—N3G	104.1 (2)
O2B—C8B—C7B	118.1 (2)	N3G—C3G—C4G	110.9 (2)
C9B—C8B—C7B	119.3 (2)	N3G—C3G—H3GA	109.5
C10B—C9B—C8B	119.5 (2)	C4G—C3G—H3GA	109.5
C10B—C9B—H9BA	120.2	N3G—C3G—H3GB	109.5
C8B—C9B—H9BA	120.2	C4G—C3G—H3GB	109.5
C9B—C10B—C5B	121.9 (2)	H3GA—C3G—H3GB	108.1
C9B—C10B—H10B	119.1	C5G—C4G—C3G	112.3 (2)
C5B—C10B—H10B	119.1	C5G—C4G—H4GA	109.1
C1B—C11B—C12B	112.9 (2)	C3G—C4G—H4GA	109.1
C1B—C11B—H11C	109.0	C5G—C4G—H4GB	109.1
C12B—C11B—H11C	109.0	C3G—C4G—H4GB	109.1
C1B—C11B—H11D	109.0	H4GA—C4G—H4GB	107.9
C12B—C11B—H11D	109.0	C6G—C5G—C10G	117.6 (2)
H11C—C11B—H11D	107.8	C6G—C5G—C4G	121.6 (2)
C11B—C12B—C13B	111.1 (2)	C10G—C5G—C4G	120.8 (2)
C11B—C12B—H12C	109.4	C7G—C6G—C5G	121.4 (2)
C13B—C12B—H12C	109.4	C7G—C6G—H6GA	119.3
C11B—C12B—H12D	109.4	C5G—C6G—H6GA	119.3
C13B—C12B—H12D	109.4	C6G—C7G—C8G	120.2 (2)
H12C—C12B—H12D	108.0	C6G—C7G—H7GA	119.9
C12B—C13B—H13D	109.5	C8G—C7G—H7GA	119.9
C12B—C13B—H13E	109.5	O2G—C8G—C9G	123.7 (2)
H13D—C13B—H13E	109.5	O2G—C8G—C7G	117.2 (2)
C12B—C13B—H13F	109.5	C9G—C8G—C7G	119.1 (2)
H13D—C13B—H13F	109.5	C8G—C9G—C10G	120.5 (2)
H13E—C13B—H13F	109.5	C8G—C9G—H9GA	119.7
N1C—C1C—N3C	111.9 (2)	C10G—C9G—H9GA	119.7
N1C—C1C—C11C	125.3 (2)	C9G—C10G—C5G	121.3 (2)
N3C—C1C—C11C	122.8 (2)	C9G—C10G—H10G	119.4
O1C—C2C—N2C	128.5 (2)	C5G—C10G—H10G	119.4
O1C—C2C—N3C	127.2 (2)	C1G—C11G—C12G	112.5 (2)
N2C—C2C—N3C	104.3 (2)	C1G—C11G—H11M	109.1
N3C—C3C—C4C	113.2 (2)	C12G—C11G—H11M	109.1
N3C—C3C—H3CA	108.9	C1G—C11G—H11N	109.1
C4C—C3C—H3CA	108.9	C12G—C11G—H11N	109.1
N3C—C3C—H3CB	108.9	H11M—C11G—H11N	107.8
C4C—C3C—H3CB	108.9	C13G—C12G—C11G	112.3 (2)
H3CA—C3C—H3CB	107.8	C13G—C12G—H12M	109.1
C5C—C4C—C3C	109.8 (2)	C11G—C12G—H12M	109.1
C5C—C4C—H4CA	109.7	C13G—C12G—H12N	109.1
C3C—C4C—H4CA	109.7	C11G—C12G—H12N	109.1
C5C—C4C—H4CB	109.7	H12M—C12G—H12N	107.9
C3C—C4C—H4CB	109.7	C12G—C13G—H13S	109.5
H4CA—C4C—H4CB	108.2	C12G—C13G—H13T	109.5
C6C—C5C—C10C	118.1 (2)	H13S—C13G—H13T	109.5
C6C—C5C—C4C	120.6 (2)	C12G—C13G—H13U	109.5
C10C—C5C—C4C	121.2 (2)	H13S—C13G—H13U	109.5
C7C—C6C—C5C	121.3 (2)	H13T—C13G—H13U	109.5
C7C—C6C—H6CA	119.3	N1H—C1H—N3H	111.3 (2)
C5C—C6C—H6CA	119.3	N1H—C1H—C11H	125.6 (3)
C6C—C7C—C8C	120.5 (2)	N3H—C1H—C11H	123.1 (2)
C6C—C7C—H7CA	119.8	O1H—C2H—N2H	128.4 (2)
C8C—C7C—H7CA	119.8	O1H—C2H—N3H	127.2 (2)
O2C—C8C—C7C	117.5 (2)	N2H—C2H—N3H	104.3 (2)
O2C—C8C—C9C	123.4 (2)	N3H—C3H—C4H	113.4 (2)
C7C—C8C—C9C	119.1 (2)	N3H—C3H—H3HA	108.9
C10C—C9C—C8C	119.9 (2)	C4H—C3H—H3HA	108.9
C10C—C9C—H9CA	120.0	N3H—C3H—H3HB	108.9
C8C—C9C—H9CA	120.0	C4H—C3H—H3HB	108.9
C9C—C10C—C5C	121.1 (2)	H3HA—C3H—H3HB	107.7
C9C—C10C—H10C	119.5	C5H—C4H—C3H	110.0 (2)
C5C—C10C—H10C	119.5	C5H—C4H—H4HA	109.7
C1C—C11C—C12C	113.7 (2)	C3H—C4H—H4HA	109.7
C1C—C11C—H11E	108.8	C5H—C4H—H4HB	109.7
C12C—C11C—H11E	108.8	C3H—C4H—H4HB	109.7
C1C—C11C—H11F	108.8	H4HA—C4H—H4HB	108.2
C12C—C11C—H11F	108.8	C10H—C5H—C6H	117.7 (2)
H11E—C11C—H11F	107.7	C10H—C5H—C4H	120.6 (3)
C11C—C12C—C13C	110.9 (2)	C6H—C5H—C4H	121.7 (2)
C11C—C12C—H12E	109.5	C7H—C6H—C5H	121.2 (2)
C13C—C12C—H12E	109.5	C7H—C6H—H6HA	119.4
C11C—C12C—H12F	109.5	C5H—C6H—H6HA	119.4
C13C—C12C—H12F	109.5	C6H—C7H—C8H	120.4 (2)
H12E—C12C—H12F	108.0	C6H—C7H—H7HA	119.8
C12C—C13C—H13G	109.5	C8H—C7H—H7HA	119.8
C12C—C13C—H13H	109.5	O2H—C8H—C9H	122.4 (2)
H13G—C13C—H13H	109.5	O2H—C8H—C7H	118.5 (2)
C12C—C13C—H13I	109.5	C9H—C8H—C7H	119.1 (2)
H13G—C13C—H13I	109.5	C8H—C9H—C10H	119.8 (2)
H13H—C13C—H13I	109.5	C8H—C9H—H9HA	120.1
N1D—C1D—N3D	111.1 (2)	C10H—C9H—H9HA	120.1
N1D—C1D—C11D	125.5 (2)	C9H—C10H—C5H	121.6 (3)
N3D—C1D—C11D	123.4 (2)	C9H—C10H—H10H	119.2
O1D—C2D—N2D	128.8 (2)	C5H—C10H—H10H	119.2
O1D—C2D—N3D	127.0 (2)	C1H—C11H—C12H	113.5 (3)
N2D—C2D—N3D	104.2 (2)	C1H—C11H—H11O	108.9
N3D—C3D—C4D	113.3 (2)	C12H—C11H—H11O	108.9
N3D—C3D—H3DA	108.9	C1H—C11H—H11P	108.9
C4D—C3D—H3DA	108.9	C12H—C11H—H11P	108.9
N3D—C3D—H3DB	108.9	H11O—C11H—H11P	107.7
C4D—C3D—H3DB	108.9	C13H—C12H—C11H	114.8 (3)
H3DA—C3D—H3DB	107.7	C13H—C12H—H12O	108.6
C5D—C4D—C3D	110.1 (2)	C11H—C12H—H12O	108.6
C5D—C4D—H4DA	109.6	C13H—C12H—H12P	108.6
C3D—C4D—H4DA	109.6	C11H—C12H—H12P	108.6
C5D—C4D—H4DB	109.6	H12O—C12H—H12P	107.5
C3D—C4D—H4DB	109.6	C12H—C13H—H13V	109.5
H4DA—C4D—H4DB	108.2	C12H—C13H—H13W	109.5
C10D—C5D—C6D	117.8 (2)	H13V—C13H—H13W	109.5
C10D—C5D—C4D	121.2 (2)	C12H—C13H—H13X	109.5
C6D—C5D—C4D	121.0 (2)	H13V—C13H—H13X	109.5
C7D—C6D—C5D	121.1 (2)	H13W—C13H—H13X	109.5
			
C1A—N1A—N2A—C2A	1.5 (3)	C8D—C9D—C10D—C5D	−0.9 (4)
C1B—N1B—N2B—C2B	−0.2 (3)	N1D—C1D—C11D—C12D	13.7 (4)
C1C—N1C—N2C—C2C	0.0 (3)	N3D—C1D—C11D—C12D	−169.0 (2)
C1D—N1D—N2D—C2D	−0.3 (3)	C1D—C11D—C12D—C13D	−174.3 (2)
C1E—N1E—N2E—C2E	0.9 (3)	N2E—N1E—C1E—N3E	0.0 (3)
C1F—N1F—N2F—C2F	−0.4 (3)	N2E—N1E—C1E—C11E	179.2 (3)
C1G—N1G—N2G—C2G	−0.1 (3)	C2E—N3E—C1E—N1E	−0.9 (3)
C1H—N1H—N2H—C2H	−1.2 (3)	C3E—N3E—C1E—N1E	−173.7 (2)
N2A—N1A—C1A—N3A	−1.0 (3)	C2E—N3E—C1E—C11E	179.9 (3)
N2A—N1A—C1A—C11A	175.8 (2)	C3E—N3E—C1E—C11E	7.1 (4)
C2A—N3A—C1A—N1A	0.2 (3)	N1E—N2E—C2E—O1E	177.7 (3)
C3A—N3A—C1A—N1A	−178.1 (2)	N1E—N2E—C2E—N3E	−1.4 (3)
C2A—N3A—C1A—C11A	−176.8 (2)	C1E—N3E—C2E—O1E	−177.7 (3)
C3A—N3A—C1A—C11A	5.0 (4)	C3E—N3E—C2E—O1E	−4.6 (4)
N1A—N2A—C2A—O1A	179.4 (3)	C1E—N3E—C2E—N2E	1.4 (3)
N1A—N2A—C2A—N3A	−1.4 (3)	C3E—N3E—C2E—N2E	174.5 (2)
C1A—N3A—C2A—O1A	180.0 (3)	C2E—N3E—C3E—C4E	105.0 (3)
C3A—N3A—C2A—O1A	−1.7 (4)	C1E—N3E—C3E—C4E	−83.3 (3)
C1A—N3A—C2A—N2A	0.7 (3)	N3E—C3E—C4E—C5E	177.8 (2)
C3A—N3A—C2A—N2A	179.1 (2)	C3E—C4E—C5E—C10E	−91.0 (3)
C2A—N3A—C3A—C4A	88.3 (3)	C3E—C4E—C5E—C6E	86.6 (3)
C1A—N3A—C3A—C4A	−93.7 (3)	C10E—C5E—C6E—C7E	−2.4 (4)
N3A—C3A—C4A—C5A	175.7 (2)	C4E—C5E—C6E—C7E	179.9 (2)
C3A—C4A—C5A—C6A	75.6 (3)	C5E—C6E—C7E—C8E	0.7 (4)
C3A—C4A—C5A—C10A	−103.2 (3)	C6E—C7E—C8E—O2E	−177.7 (3)
C10A—C5A—C6A—C7A	0.7 (4)	C6E—C7E—C8E—C9E	1.4 (4)
C4A—C5A—C6A—C7A	−178.1 (2)	O2E—C8E—C9E—C10E	177.3 (2)
C5A—C6A—C7A—C8A	−0.3 (4)	C7E—C8E—C9E—C10E	−1.7 (4)
C6A—C7A—C8A—O2A	−179.4 (2)	C8E—C9E—C10E—C5E	0.0 (4)
C6A—C7A—C8A—C9A	0.0 (4)	C6E—C5E—C10E—C9E	2.0 (4)
O2A—C8A—C9A—C10A	179.2 (3)	C4E—C5E—C10E—C9E	179.7 (2)
C7A—C8A—C9A—C10A	−0.2 (4)	N1E—C1E—C11E—C12E	12.2 (4)
C8A—C9A—C10A—C5A	0.7 (4)	N3E—C1E—C11E—C12E	−168.7 (3)
C6A—C5A—C10A—C9A	−0.9 (4)	C1E—C11E—C12E—C13E	72.5 (4)
C4A—C5A—C10A—C9A	177.9 (2)	N2F—N1F—C1F—N3F	−0.4 (3)
N1A—C1A—C11A—C12A	6.5 (4)	N2F—N1F—C1F—C11F	178.8 (2)
N3A—C1A—C11A—C12A	−177.0 (2)	C2F—N3F—C1F—N1F	1.1 (3)
C1A—C11A—C12A—C13A	178.3 (3)	C3F—N3F—C1F—N1F	−179.9 (2)
N2B—N1B—C1B—N3B	−0.9 (3)	C2F—N3F—C1F—C11F	−178.1 (2)
N2B—N1B—C1B—C11B	179.4 (2)	C3F—N3F—C1F—C11F	0.9 (4)
C2B—N3B—C1B—N1B	1.6 (3)	N1F—N2F—C2F—O1F	−178.6 (3)
C3B—N3B—C1B—N1B	179.9 (2)	N1F—N2F—C2F—N3F	1.1 (3)
C2B—N3B—C1B—C11B	−178.6 (2)	C1F—N3F—C2F—O1F	178.4 (3)
C3B—N3B—C1B—C11B	−0.4 (4)	C3F—N3F—C2F—O1F	−0.7 (4)
N1B—N2B—C2B—O1B	−179.2 (3)	C1F—N3F—C2F—N2F	−1.2 (3)
N1B—N2B—C2B—N3B	1.1 (3)	C3F—N3F—C2F—N2F	179.7 (2)
C1B—N3B—C2B—O1B	178.7 (3)	C2F—N3F—C3F—C4F	91.5 (3)
C3B—N3B—C2B—O1B	0.4 (4)	C1F—N3F—C3F—C4F	−87.4 (3)
C1B—N3B—C2B—N2B	−1.6 (3)	N3F—C3F—C4F—C5F	175.8 (2)
C3B—N3B—C2B—N2B	−180.0 (2)	C3F—C4F—C5F—C6F	97.0 (3)
C1B—N3B—C3B—C4B	−89.3 (3)	C3F—C4F—C5F—C10F	−82.3 (3)
C2B—N3B—C3B—C4B	88.7 (3)	C10F—C5F—C6F—C7F	−1.5 (4)
N3B—C3B—C4B—C5B	179.1 (2)	C4F—C5F—C6F—C7F	179.2 (3)
C3B—C4B—C5B—C6B	99.4 (3)	C5F—C6F—C7F—C8F	0.0 (5)
C3B—C4B—C5B—C10B	−80.9 (3)	C6F—C7F—C8F—O2F	−178.4 (3)
C10B—C5B—C6B—C7B	−3.1 (4)	C6F—C7F—C8F—C9F	1.6 (5)
C4B—C5B—C6B—C7B	176.6 (2)	O2F—C8F—C9F—C10F	178.3 (3)
C5B—C6B—C7B—C8B	1.2 (4)	C7F—C8F—C9F—C10F	−1.7 (4)
C6B—C7B—C8B—O2B	−179.3 (2)	C6F—C5F—C10F—C9F	1.4 (4)
C6B—C7B—C8B—C9B	1.6 (4)	C4F—C5F—C10F—C9F	−179.3 (3)
O2B—C8B—C9B—C10B	178.7 (2)	C8F—C9F—C10F—C5F	0.2 (4)
C7B—C8B—C9B—C10B	−2.3 (4)	N1F—C1F—C11F—C12F	0.9 (4)
C8B—C9B—C10B—C5B	0.2 (4)	N3F—C1F—C11F—C12F	−180.0 (2)
C6B—C5B—C10B—C9B	2.5 (4)	C1F—C11F—C12F—C13F	73.6 (3)
C4B—C5B—C10B—C9B	−177.3 (2)	N2G—N1G—C1G—N3G	0.7 (3)
N1B—C1B—C11B—C12B	0.4 (4)	N2G—N1G—C1G—C11G	−179.0 (2)
N3B—C1B—C11B—C12B	−179.3 (2)	C2G—N3G—C1G—N1G	−1.1 (3)
C1B—C11B—C12B—C13B	179.6 (3)	C3G—N3G—C1G—N1G	−177.6 (2)
N2C—N1C—C1C—N3C	0.0 (3)	C2G—N3G—C1G—C11G	178.6 (2)
N2C—N1C—C1C—C11C	−177.7 (2)	C3G—N3G—C1G—C11G	2.2 (4)
C2C—N3C—C1C—N1C	0.1 (3)	N1G—N2G—C2G—O1G	179.5 (3)
C3C—N3C—C1C—N1C	178.4 (2)	N1G—N2G—C2G—N3G	−0.6 (3)
C2C—N3C—C1C—C11C	177.8 (2)	C1G—N3G—C2G—O1G	−179.1 (3)
C3C—N3C—C1C—C11C	−3.9 (4)	C3G—N3G—C2G—O1G	−2.5 (4)
N1C—N2C—C2C—O1C	−179.2 (2)	C1G—N3G—C2G—N2G	1.0 (3)
N1C—N2C—C2C—N3C	0.1 (3)	C3G—N3G—C2G—N2G	177.6 (2)
C1C—N3C—C2C—O1C	179.2 (3)	C2G—N3G—C3G—C4G	−97.2 (3)
C3C—N3C—C2C—O1C	0.8 (4)	C1G—N3G—C3G—C4G	78.7 (3)
C1C—N3C—C2C—N2C	−0.1 (3)	N3G—C3G—C4G—C5G	−178.1 (2)
C3C—N3C—C2C—N2C	−178.5 (2)	C3G—C4G—C5G—C6G	−101.3 (3)
C2C—N3C—C3C—C4C	−91.2 (3)	C3G—C4G—C5G—C10G	79.2 (3)
C1C—N3C—C3C—C4C	90.7 (3)	C10G—C5G—C6G—C7G	0.3 (4)
N3C—C3C—C4C—C5C	−177.6 (2)	C4G—C5G—C6G—C7G	−179.3 (3)
C3C—C4C—C5C—C6C	−71.9 (3)	C5G—C6G—C7G—C8G	−0.2 (4)
C3C—C4C—C5C—C10C	105.1 (3)	C6G—C7G—C8G—O2G	−179.4 (3)
C10C—C5C—C6C—C7C	−1.3 (4)	C6G—C7G—C8G—C9G	0.1 (4)
C4C—C5C—C6C—C7C	175.7 (2)	O2G—C8G—C9G—C10G	179.5 (2)
C5C—C6C—C7C—C8C	0.2 (4)	C7G—C8G—C9G—C10G	0.0 (4)
C6C—C7C—C8C—O2C	−178.0 (2)	C8G—C9G—C10G—C5G	0.0 (4)
C6C—C7C—C8C—C9C	1.5 (4)	C6G—C5G—C10G—C9G	−0.1 (4)
O2C—C8C—C9C—C10C	177.4 (2)	C4G—C5G—C10G—C9G	179.4 (2)
C7C—C8C—C9C—C10C	−2.1 (4)	N1G—C1G—C11G—C12G	−8.5 (4)
C8C—C9C—C10C—C5C	1.0 (4)	N3G—C1G—C11G—C12G	171.8 (2)
C6C—C5C—C10C—C9C	0.7 (4)	C1G—C11G—C12G—C13G	−73.1 (3)
C4C—C5C—C10C—C9C	−176.3 (2)	N2H—N1H—C1H—N3H	1.0 (3)
N1C—C1C—C11C—C12C	1.9 (4)	N2H—N1H—C1H—C11H	−178.4 (3)
N3C—C1C—C11C—C12C	−175.5 (2)	C2H—N3H—C1H—N1H	−0.5 (3)
C1C—C11C—C12C—C13C	177.0 (3)	C3H—N3H—C1H—N1H	174.0 (2)
N2D—N1D—C1D—N3D	−0.9 (3)	C2H—N3H—C1H—C11H	178.9 (3)
N2D—N1D—C1D—C11D	176.7 (2)	C3H—N3H—C1H—C11H	−6.6 (4)
C2D—N3D—C1D—N1D	1.7 (3)	N1H—N2H—C2H—O1H	−178.3 (3)
C3D—N3D—C1D—N1D	−172.5 (2)	N1H—N2H—C2H—N3H	0.9 (3)
C2D—N3D—C1D—C11D	−176.0 (2)	C1H—N3H—C2H—O1H	179.0 (3)
C3D—N3D—C1D—C11D	9.9 (4)	C3H—N3H—C2H—O1H	4.2 (4)
N1D—N2D—C2D—O1D	−179.2 (3)	C1H—N3H—C2H—N2H	−0.3 (3)
N1D—N2D—C2D—N3D	1.2 (3)	C3H—N3H—C2H—N2H	−175.0 (2)
C1D—N3D—C2D—O1D	178.8 (3)	C2H—N3H—C3H—C4H	−98.9 (3)
C3D—N3D—C2D—O1D	−6.9 (4)	C1H—N3H—C3H—C4H	87.5 (3)
C1D—N3D—C2D—N2D	−1.7 (3)	N3H—C3H—C4H—C5H	−172.2 (2)
C3D—N3D—C2D—N2D	172.7 (2)	C3H—C4H—C5H—C10H	89.9 (3)
C2D—N3D—C3D—C4D	100.8 (3)	C3H—C4H—C5H—C6H	−87.0 (3)
C1D—N3D—C3D—C4D	−86.0 (3)	C10H—C5H—C6H—C7H	4.1 (4)
N3D—C3D—C4D—C5D	176.0 (2)	C4H—C5H—C6H—C7H	−178.9 (3)
C3D—C4D—C5D—C10D	−110.4 (3)	C5H—C6H—C7H—C8H	−1.7 (4)
C3D—C4D—C5D—C6D	69.8 (3)	C6H—C7H—C8H—O2H	178.4 (3)
C10D—C5D—C6D—C7D	0.4 (4)	C6H—C7H—C8H—C9H	−1.8 (4)
C4D—C5D—C6D—C7D	−179.8 (2)	O2H—C8H—C9H—C10H	−177.5 (3)
C5D—C6D—C7D—C8D	−0.2 (4)	C7H—C8H—C9H—C10H	2.7 (4)
C6D—C7D—C8D—O2D	179.8 (3)	C8H—C9H—C10H—C5H	−0.2 (4)
C6D—C7D—C8D—C9D	−0.6 (4)	C6H—C5H—C10H—C9H	−3.2 (4)
O2D—C8D—C9D—C10D	−179.3 (2)	C4H—C5H—C10H—C9H	179.8 (3)
C7D—C8D—C9D—C10D	1.1 (4)	N1H—C1H—C11H—C12H	−16.3 (5)
C6D—C5D—C10D—C9D	0.2 (4)	N3H—C1H—C11H—C12H	164.4 (3)
C4D—C5D—C10D—C9D	−179.6 (2)	C1H—C11H—C12H—C13H	−67.5 (4)

### Hydrogen-bond geometry (Å, º)

**Table:**

*D*—H···*A*	*D*—H	H···*A*	*D*···*A*	*D*—H···*A*
O2*A*—H2*A*···O1*H*	0.82	1.90	2.718 (3)	174
O2*C*—H2*C*···O1*E*	0.82	1.90	2.717 (3)	172
O2*G*—H2*G*···O1*C*	0.82	1.89	2.701 (3)	172
O2*H*—H2*H*···O1*D*	0.82	1.94	2.752 (3)	174
N2*B*—H2*BA*···O1*C*	0.86	1.97	2.821 (3)	169
N2*C*—H2*CA*···O1*B*	0.86	1.97	2.825 (3)	174
N2*F*—H2*FA*···O1*H*	0.86	1.93	2.777 (3)	166
N2*H*—H2*HA*···O1*F*	0.86	2.00	2.840 (3)	167
O2*B*—H2*B*···O1*G*^i^	0.82	1.93	2.748 (3)	177
O2*D*—H2*D*···O1*F*^i^	0.82	1.87	2.689 (3)	178
O2*E*—H2*E*···O1*B*^ii^	0.82	1.97	2.773 (3)	165
O2*F*—H2*F*···O1*A*^ii^	0.82	1.92	2.711 (3)	162
N2*A*—H2*AA*···O1*D*^iii^	0.86	1.94	2.788 (3)	170
N2*E*—H2*EA*···O1*G*^iii^	0.86	2.03	2.873 (3)	168
N2*D*—H2*DA*···O1*A*^iv^	0.86	1.95	2.809 (3)	173
N2*G*—H2*GA*···O1*E*^iv^	0.86	1.96	2.807 (3)	168

Symmetry codes: (i) *x*−1/2, *y*+1/2, *z*; (ii) *x*+1/2, *y*−1/2, *z*; (iii) *x*+1/2, *y*+1/2, *z*; (iv) *x*−1/2, *y*−1/2, *z*.
